# Engineered basement membrane mimetic hydrogels to study mammary epithelial morphogenesis and invasion

**DOI:** 10.1126/sciadv.adx2110

**Published:** 2025-09-26

**Authors:** Jane A. Baude, Megan D. Li, Sabrina M. Jackson, Abhishek Sharma, Daniella I. Walter, Ryan S. Stowers

**Affiliations:** ^1^ Department of Molecular, Cellular, and Developmental Biology, University of California, Santa Barbara, Santa Barbara, CA 93106, USA.; ^2^ Department of Mechanical Engineering, University of California, Santa Barbara, Santa Barbara, CA 93106, USA.; ^3^ Department of Bioengineering, University of California, Santa Barbara, Santa Barbara, CA 93106, USA.

## Abstract

Reconstituted basement membrane products, like Matrigel, suffer from variability and xenogenic contaminants, hindering three-dimensional cell culture models. To overcome these challenges, we developed engineered basement membranes (eBMs) using peptide-conjugated alginate hydrogels with independently tunable mechanics. Ile-Lys-Val-Ala-Val (IKVAV)–modified eBMs, with fast stress relaxation and low stiffness, supported normal mammary acinus formation. Both increased stiffness and slow relaxation were required to induce invasion in IKVAV-modified eBMs, differing from the invasive phenotype observed in Arg-Gly-Asp (RGD)–modified eBMs regardless of the mechanical properties. Mechanistic studies revealed the balance of β1 and β4 integrin signaling, hemidesmosome formation, and laminin production were influenced by eBM properties. Inhibiting focal adhesion kinase or hemidesmosome signaling disrupted acinus formation in IKVAV-modified eBMs. This defined, xenogenic-free eBM system offers a modular platform for tissue engineering and disease modeling.

## Teaser

A modular, tunable platform helps unravel cell-matrix interactions and drives tissue growth.

## INTRODUCTION

The three-dimensional (3D) microenvironment surrounding the mammary epithelium profoundly influences both the normal morphogenesis of these cells and progression of breast tumors (*1*–*5*). Throughout development and into adulthood, the mammary epithelium undergoes continuous and dynamic morphogenetic changes, often accompanied by substantial remodeling of the extracellular matrix (ECM). Alterations in ECM composition and mechanical properties play a critical role in modulating these morphogenetic processes, including epithelial-to-mesenchymal transition, and are key drivers of tumor progression (*6*–*8*). However, because of the ECM’s complexity and its dynamic, interactive components, fully understanding the interplay between ECM behavior and morphogenetic programs requires the development of a controllable and tunable in vitro platform.

In vivo, basement membrane proteins of the ECM directly contact mammary epithelium cells (MECs) and govern their phenotype and polarization. Most in vitro models of mammary morphogenesis rely on reconstituted basement membrane (rBM) extract (i.e., Matrigel, Cultrex, and Geltrex) to recapitulate the epithelial basement membrane (*9*). Researchers first established the use of 3D rBM culture for MECs in studies that emphasized the important role of rBM in facilitating mammary morphogenesis in vitro (*1*, *10*). As a result, the rBM has become a cornerstone for investigating breast cancer mechanobiology, with landmark studies (*11*–*18*) demonstrating how the mechanical properties of the ECM can drive tumor progression. The tumor microenvironment is significantly stiffer than healthy tissue, and studies have shown that matrix stiffness is a key mechanical property that enhances the tumorigenic behavior of mammary epithelial cells in rBM systems (*18*, *19*). 3D in vitro studies have shown that kilopascal stiffness induces malignant phenotypes in MECs, while soft substrates (<200 Pa) support a nonmalignant mammary acinar phenotype (*17*). While the literature extensively documents the impacts of matrix stiffness in vivo and in vitro (*17*, *18*, *20*–*22*), viscoelasticity has received less attention in the context of the mammary epithelium, although recent studies demonstrate that viscoelasticity can affect mammary epithelial and breast cancer cell phenotypes (*23*–*25*). Although the rBM is inherently viscoelastic, the field has only recently started to investigate these properties within 3D culture systems (*26*–*31*).

Despite its widespread use, rBM products suffer from several limitations, including batch-to-batch variability, a lack of mechanical and biochemical tunability, the presence of undefined growth factors and signaling molecules, xenogenic contaminants, and recently, pandemic-related supply shortages (*32*–*34*). These limitations have driven the development of engineered matrices that mimic key features of rBM. Hydrogels, particularly those derived from bioinert materials, offer a versatile platform. While engineered rBM-free systems have shown promise in other organoid models, such as intestinal and neuroepithelial models (*35*–*38*), fully recapitulating mammary morphogenesis without the rBM remains a substantial challenge. Deciphering the mechanisms by which biochemical and mechanical cues, both independently and in concert, can influence mammary epithelial cell behavior is crucial to achieve this goal. This underscores a twofold critical need: first, to develop an rBM-free system that effectively supports mammary morphogenesis and, second, to ensure that the system is defined and tunable, which allows for a systematic exploration of the interplay between mechanical and biochemical cues.

In this study, we endeavored to develop a defined, engineered rBM-free matrix system to promote MEC acinar morphogenesis and invasion in vitro. To achieve this, we conjugated a panel of basement membrane–derived adhesion motifs to a mechanically tunable alginate hydrogel network. This innovative, bottom-up approach allows us to precisely and independently manipulate both the biochemical and mechanical attributes of the matrix. By encapsulating MECs within these matrices, we gain a clearer understanding of how the distinct biochemical and mechanical properties influence the MEC phenotype individually. Our platform offers critical insights into mammary epithelial cell adhesion and morphogenesis, mechanotransduction, and basement membrane production that traditional rBM systems often overlook, illuminating the complex interplay of cellular behavior within a finely controlled environment.

## RESULTS

### Alginate networks can be mechanically and biochemically tuned

**Fig. 1.**
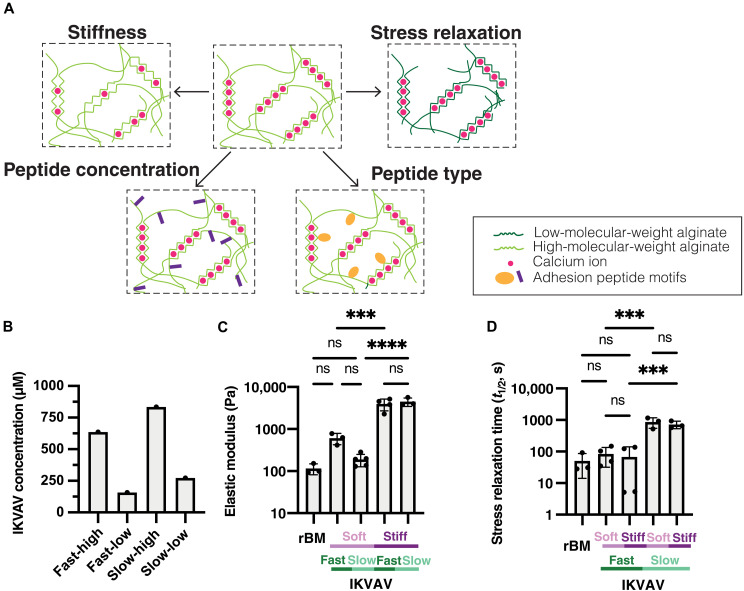
Modified eBMs have independently tunable mechanical and biochemical properties. (**A**) Alginate-based eBMs have tunable mechanical and biochemical properties. Stiffness can be modulated by adjusting the concentration of calcium for cross-linking. Stress relaxation can be changed by using alginates of different molecular weights. Peptides of different types or concentrations can be conjugated to the alginate network. (**B**) Summary of the concentration of the IKVAV peptide on modified fast-relaxing and slow-relaxing alginate from H NMR data (fig. S1 and table S1). (**C**) Elastic modulus of rBM and IKVAV-modified eBMs. (**D**) Stress relaxation rate of rBM and IKVAV-modified eBMs. Significance was determined by a one-way analysis of variance (ANOVA) for elastic modulus and stress relaxation time (*P* value <0.05). *n* = 1 replicate per condition for H NMR measurements. *n* = 3 or 4 replicates for elastic modulus and stress relaxation time. ***P < 0.001 and ****P < 0.0001; ns, not significant.

Using an alginate-based system, matrix stiffness, stress relaxation rate, and adhesion ligand type and density can be tuned independently of each other without altering the matrix pore size and architecture (Fig. 1A) (*39*). Alginate presents no cell adhesion motifs recognized by mammalian cells; thus, cell adhesion cues can be entirely controlled by conjugating synthetic adhesion peptide sequences to the alginate chains. Laminin-111 is one of the most abundant and critical proteins found in the mammary basement membrane and in rBM products (*40*–*45*). Levels of laminin-111 fluctuate significantly during mammary morphogenesis and the progression of breast cancer (*46*, *47*), suggesting that laminin-111 adhesion motifs are promising candidates for modeling the basement membrane. Thus, we selected the laminin-111 adhesion motifs Ile-Lys-Val-Ala-Val (IKVAV) and Tyr-Ile-Gly-Ser-Arg (YIGSR) to minimally mimic key motifs of the basement membrane and rBM (*48*). As a point of comparison, we also selected an Arg-Gly-Asp (RGD) motif, which is primarily associated with fibronectin but also found on several ECM proteins, including laminin-111 (*49*). In addition, RGD adhesion peptides have been extensively used in engineered hydrogel platforms to promote cell adhesion (*14*, *50*–*56*), which are often compared to rBM products (*33*, *57*, *58*). We used carbodiimide chemistry (*49*) to couple peptide sequences containing the adhesion motifs IKVAV, YIGSR, or RGD to purified alginate, at both high and low concentrations, to test the impacts of changing ligand density (Fig. 1, A and B). We verified peptide conjugation via H nuclear magnetic resonance (NMR) (figs. S1 and S2 and table S1).

Matrix stiffness, or the resistance to deformation, is known to affect the MCF10A phenotype in 3D cell culture studies (*14*, *17*) . Therefore, it is crucial to have independent control of matrix stiffness relative to other mechanical properties like stress relaxation or pore size within this system. The cross-linking density, and thus stiffness, of alginate hydrogels is a function of the concentration of divalent cations, such as Ca^2+^, used to generate ionic cross-links. We optimized calcium concentrations for peptide-modified alginate matrices to mimic the reported elastic moduli of either normal mammary gland tissue (<500 Pa, “soft”) or primary breast tumors (4000 Pa, “stiff”) (Fig. 1C and fig. S3) (*59*).

We measured the stress relaxation half-time (τ_1/2_) of rBM to be ~20 to 90 s, indicating that it is highly viscoelastic (Fig. 1D). To vary stress relaxation rates in alginate matrices, we used alginates of different molecular weights (*39*). Low-molecular-weight alginate modified with IKVAV yielded similar stress relaxation profiles to the rBM (τ_1/2_ = 30 to 150 s) (Fig. 1D). In contrast, high-molecular-weight alginate modified with IKVAV produced slow-relaxing matrices (τ_1/2_ = 600 to 1100 s) (Fig. 1D) (*39*). The elastic modulus and stress relaxation rate can be independently tuned (Fig. 1, C and D, and fig. S3). We found similar results with YIGSR alginate (fig. S3, H and I). Statistical analysis showed no significant differences in stiffness between matrices with different stress relaxation rates nor in stress relaxation rates between matrices of different stiffness. Thus, both soft and stiff matrices can be fabricated with either fast or slow stress relaxation for both IKVAV- and YIGSR-modified engineered basement membranes (eBMs) (Fig. 1, C and D, and fig. S3, H and I).

### Soft, fast-relaxing modified matrices enable mammary acinus morphogenesis without the rBM

Mammary acini are 3D structures that develop within a 7- to 14-day period, distinguished by their apical-basal polarity and hollow lumens (*1*). MCF10A breast epithelial cells encapsulated in the rBM develop into polarized acini, mimicking in vivo mammary acini (*10*). To assess whether the acinar morphogenesis of MCF10A cells could occur in modified eBMs, we encapsulated MCF10A cell matrices that most closely mimicked the properties of rBM, namely a high concentration of adhesion peptides, fast relaxation, and soft modulus. Within these soft, fast-relaxing matrices, we varied the peptide motifs to create three distinct conditions: IKVAV-, YIGSR-, and RGD-modified eBMs.

**Fig. 2.**
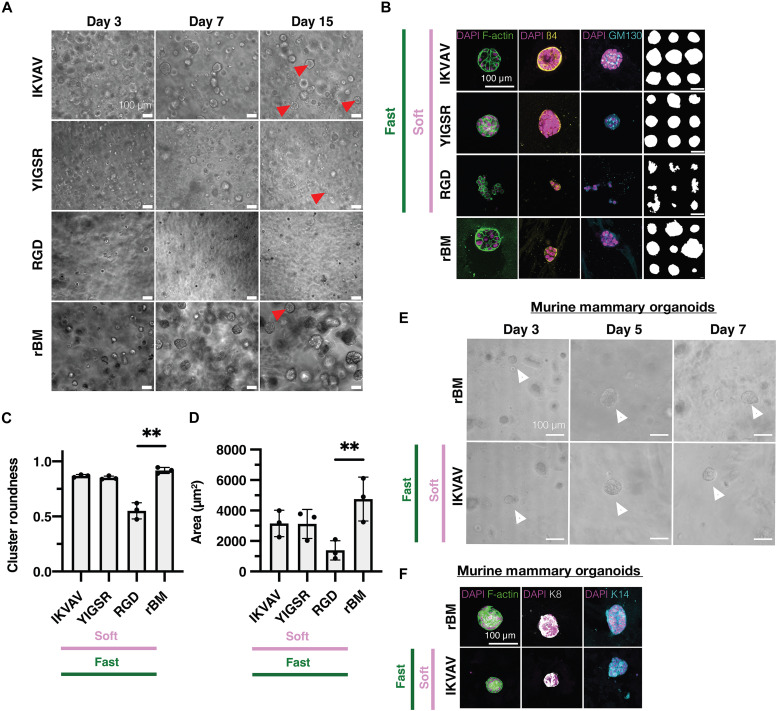
Modified alginate eBMs mimicking the rBM enable mammary acinus formation and organoid morphogenesis without the rBM. (**A**) Representative bright-field images of MCF10A cells in soft, fast-relaxing eBMs, modified with either IKVAV, YIGSR, or RGD, in comparison to MCF10A cells grown in the rBM. Images are from days 3, 7, and 15 during culture. Red arrowheads indicate examples of acinar-like clusters. (**B**) Representative confocal images of MCF10A cells in soft, fast-relaxing eBMs, modified with either IKVAV, YIGSR, or RGD, compared to MCF10A cells encapsulated in the rBM. From left to right: DAPI/F-actin, DAPI/β4 integrin, DAPI/GM130, and representative outlines of the respective condition. Scale bars, 100 μm. (**C**) Quantification of cluster roundness from the soft, fast-relaxing eBMs. (**D**) Quantification of cluster area from the soft, fast-relaxing eBMs. (**E**) Representative bright-field images of mammary murine organoids in soft, fast-relaxing, IKVAV-modified eBMs and the rBM. (**F**) Representative confocal images of mammary murine organoids. From left to right: DAPI/F-actin, DAPI/keratin-8, and DAPI/keratin-14. All scale bars, 100 μm. Data are shown as the means ± SD of three biological replicates (*n* = 3, average of 20 to 50 images per replicate), unless otherwise indicated. Significance was determined by a Kruskal-Wallis test followed by Dunnett’s multiple testing correction for MCF10A cluster roundness and by a one-way ANOVA followed by post hoc multiple comparison tests for cluster area. An unpaired *t* test was used for organoid cluster roundness. If no statistical significance indicator bars are shown, there were no significant differences (*P* value >0.05). **P < 0.01.

The cells were cultured in the respective matrix condition for 2 weeks, and then the cell cluster morphology was analyzed and compared to cells cultured in the rBM. In soft, IKVAV-modified eBMs, MCF10A cells developed into acinar-like clusters over the 2-week culture period, very similar to cells grown in the rBM (Fig. 2A). MCF10A cells cultured in YIGSR-modified eBMs also formed round clusters that closely resembled those observed in the rBM, although with less frequency than in IKVAV-modified matrices (Fig. 2, A and C). In contrast, MCF10A cells encapsulated in soft, RGD-modified matrices remained as single cells or small, irregularly shaped clusters (Fig. 2A). To further validate the extent of acinus formation and maturation in these matrices, we performed immunostaining and confocal microscopy for key markers of polarization: β4 integrin (basal) and Golgi matrix protein 130 (GM130, apical). In soft, fast-relaxing, IKVAV-modified eBMs, we observed similar polarization to that of cell clusters in the rBM (Fig. 2B). For instance, β4 integrin localized to the basement membrane in both the IKVAV-modified matrix and rBM, while GM130 was apical to the nuclei of the cluster under both conditions (Fig. 2B and fig. S4, A and B). Of note, in soft, fast-relaxing, YIGSR-modified eBMs, polarization was less pronounced than in IKVAV-modified matrices despite the rounded clusters (Fig. 2, B and C). In the RGD-modified matrices, both β4 integrin and GM130 were distributed throughout the cluster, indicating a lack of polarization (Fig. 2B).

We also analyzed the overall cell cluster roundness and area. MCF10A clusters in soft, IKVAV- and YIGSR-modified eBMs had high roundness values, which were not significantly different from each other or from those measured from clusters in the rBM (Fig. 2C). In contrast, clusters in the soft, RGD-modified eBMs were significantly less round (Fig. 2C). We also compared the sizes of the different clusters. Clusters grown in IKVAV- or YIGSR-modified matrices had similar area to those grown in the rBM, while clusters in RGD-modified matrices were significantly smaller (Fig. 2D).

To assess the utility of this system as an alternative to the rBM for other cell culture studies, we next attempted to culture both murine mammary organoids and patient-derived organoids in modified matrices. We chose to compare soft, fast-relaxing, IKVAV-modified matrices against the rBM for these different types of organoids because this condition generated the most robust acinus formation with MCF10A cells. Murine mammary organoids grown in soft, fast-relaxing, IKVAV-modified eBMs grew and formed round structures, similar to those seen in the rBM (Fig. 2, E and F). Keratin-8, a luminal cell marker, was abundant within the center of these structures, while keratin-14, a basal cell marker, was more intense at the basal membrane of these structures (*60*). The localization of these keratin markers was similar between IKVAV-modified alginate matrices and the rBM and is also consistent with a prior report of mammary organoids in growth in synthetic matrices (*61*). Excitingly, patient-derived PDM-350 organoids grown in soft, fast-relaxing, IKVAV-modified eBMs were very round (fig. S5, A and B) and not significantly different in cluster roundness from those grown in rBM matrices (fig. S5B). These results suggest that soft, fast-relaxing, IKVAV-modified eBMs provide a suitable environment for organoids, supporting their growth and morphology similarly to traditional rBM matrices.

### Tumor-like matrix stiffness alone does not induce an invasive phenotype

**Fig. 3.**
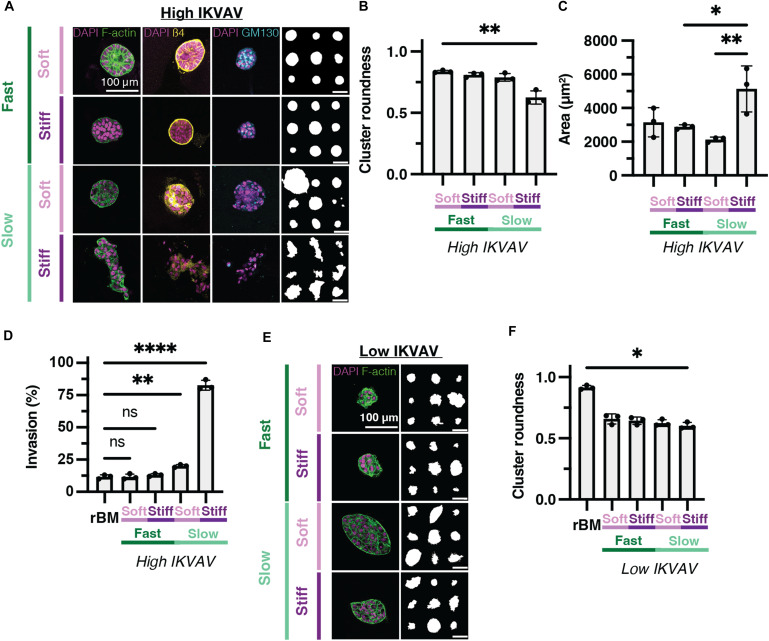
Both elevated stiffness and slow stress relaxation are required to induce invasion in IKVAV-modified eBMs. (**A**) Representative confocal images of MCF10A cells encapsulated in soft, fast-relaxing; stiff, fast-relaxing; soft, slow-relaxing; and stiff, slow-relaxing, high-concentration IKVAV–modified eBMs. From left to right: DAPI/F-actin, DAPI/β4 integrin, DAPI/GM130, and representative outlines of the respective condition. (**B**) Quantification of cluster roundness for high-concentration IKVAV–modified eBMs. (**C**) Quantification of cluster area for IKVAV-modified eBMs. (**D**) Quantification of the percentage of invasive clusters in IKVAV-modified eBMs. (**E**) Representative confocal images of MCF10A cells encapsulated in soft, fast-relaxing; stiff, fast-relaxing; soft, slow-relaxing; and stiff, slow-relaxing, low-concentration IKVAV–modified eBMs. From left to right: DAPI/F-actin and representative outlines. (**F**) Quantification of cluster roundness for low-concentration IKVAV–modified eBMs. All scale bars, 100 μm. Data are shown as the means ± SD of three biological replicates (*n* = 3, 20 to 50 images per replicate), unless otherwise indicated. Statistical significance was tested by a Kruskal-Wallis test followed by Dunnett’s multiple testing correction for cluster roundness and percentage of invasive clusters and by a one-way ANOVA and post hoc multiple comparison tests for cluster area. If no statistical significance indicator bars are shown, there were no significant differences (*P* value >0.05). *P < 0.05, **P < 0.01, and ****P < 0.0001.

After demonstrating that modified matrices could support mammary acinar morphogenesis without the rBM in soft, fast-relaxing, IKVAV-modified matrices, we sought to use the mechanical tunability of this platform to investigate how changes in stiffness or stress relaxation would affect the phenotype of MCF10A cells. Matrix stiffness in the range of 1 to 5 kPa is known to induce a tumorigenic phenotype in MCF10A cells, and we thus hypothesized that modified eBMs with elevated stiffness would drive invasion (*17*, *62*). Unexpectedly, in the fast-relaxing, IKVAV-modified matrices, we found no significant differences in the cluster area, roundness, or invasiveness of MCF10A cells between the soft and stiff matrices (Fig. 3, A to D). Under both conditions, acinar development was evident, characterized by clear apical-basal polarity and high roundness (Fig. 3, A and B).

Although it is established that different tissues have distinct stress relaxation properties (*39*) and specifically that breast tumors stress relax at different rates than normal mammary tissue (*24*), the precise impact of stress relaxation on mammary acinar morphogenesis remains unclear. Therefore, we encapsulated MCF10A cells in slow-relaxing, IKVAV-modified matrices to compare to fast-relaxing, IKVAV-modified eBMs. In soft, slow-relaxing, IKVAV-modified matrices, we observed round clusters (Fig. 3, A and B), yet these clusters were marked by increased β4 integrin within the cluster as opposed to localizing to the basement membrane, indicating a loss of polarization (Fig. 3A). Notably, when the IKVAV-modified eBMs were both stiff and slow relaxing, we observed the emergence of multiple invasive regions, loss of polarity and roundness, and increased cluster size (Fig. 3, A and D), consistent with prior reports of elevated stiffness driving MCF10A invasion. Cluster roundness decreased significantly, and the cluster area increased significantly over the 2-week experiment (fig. S6, A to C). Furthermore, stiff, slow-relaxing, IKVAV-modified matrices had significantly more invasive clusters in comparison to the three other IKVAV conditions tested (Fig. 3D). Thus, in IKVAV-modified eBMs, a stiff and slow-relaxing matrix is essential to promote the invasive phenotype. Notably, we observed no noticeable formation of mammary acinar structures in eBMs modified with a low concentration of IKVAV across the mechanical conditions tested. While these clusters were not invasive, they exhibited lower cluster roundness than cells cultured in the rBM and were generally more disorganized compared to cells in either the rBM or high-concentration IKVAV matrices (Fig. 3, E and F).

MCF10A cells encapsulated in soft, fast-relaxing, YIGSR-modified eBMs developed acinar-like clusters, but these clusters were less uniform in size and shape and showed less defined lumen formation (fig. S3, A to C) compared to those formed in soft, fast-relaxing, IKVAV-modified eBMs. We also encapsulated MCF10A cells in YIGSR-modified eBMs that were fast relaxing and stiff, as well as slow relaxing and soft and slow relaxing and stiff (fig. S3, A to C). We did not see consistent acinar or invasive phenotypes and therefore did not investigate these eBMs further. In addition, we did not observe any significant mammary morphogenesis in low-concentration YIGSR–modified eBMs across the mechanical conditions tested (fig. S3, D to F).

### RGD-modified matrices induce aberrant phenotypes and invasion, independent of mechanical properties

**Fig. 4.**
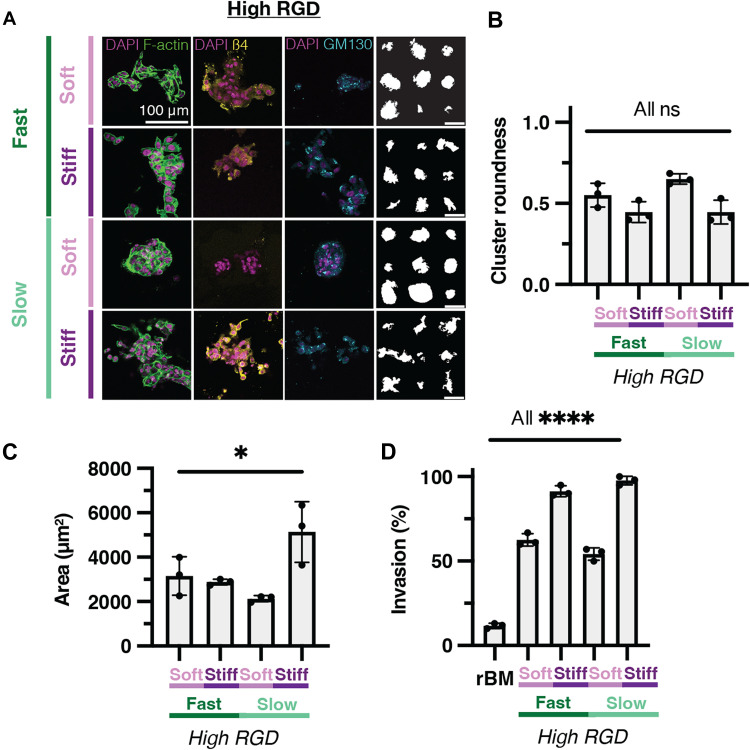
RGD-modified matrices induce aberrant phenotypes and invasion, independent of mechanical properties. (**A**) Representative confocal images of MCF10A cells encapsulated in soft, fast-relaxing; stiff, fast-relaxing; soft, slow-relaxing; and stiff, slow-relaxing, high-concentration RGD–modified eBMs. From left to right: DAPI/F-actin, DAPI/β4 integrin, DAPI/GM130, and representative outlines of the respective condition. (**B**) Quantification of cluster roundness for high-concentration RGD–modified eBMs. (**C**) Quantification of cluster area for RGD-modified eBMs. (**D**) Quantification of the percentage of invasive clusters in RGD-modified eBMs. All scale bars, 100 μm. Data are shown as the means ± SD of three biological replicates (*n* = 3, 20 to 50 images per replicate), unless otherwise indicated. Statistical significance was determined by a Kruskal-Wallis test followed by Dunnett’s multiple testing correction for cluster roundness and the percentage of invasive clusters and by a one-way ANOVA and post hoc multiple comparison tests for cluster area. If no statistical significance indicator bars are shown, there were no significant differences (*P* value >0.05). *P < 0.05 and ****P < 0.0001.

We next sought to understand how MCF10A cells behave in RGD-modified matrices. Notably, MCF10A cells grown in RGD-modified eBMs did not form acini under any conditions, underscoring the necessity of appropriate adhesion ligands for acinar development. We saw invasive phenotypes in all combinations of mechanical properties (Fig. 4A and fig. S6A). Neither β4 integrin nor GM130 localized to the basal or apical membrane, respectively, in MCF10A clusters in RGD-modified eBMs (Fig. 4A), indicating a lack of polarization. MCF10A cluster roundness nor area was significantly different between mechanical conditions of RGD-modified eBMs tested (Fig. 4, B and C). Over the course of 2 weeks, cluster roundness decreased significantly, and the area increased significantly for all RGD-modified eBMs (fig. S6, A to C). Furthermore, the percentage of invasive clusters in RGD-modified eBMs was significantly higher than those found in the rBM, with the stiff, slow-relaxing, RGD-modified group being the most invasive (Fig. 4D).

### RGD addition does not enhance acinus formation in IKVAV- or YIGSR-modified eBMs

RGD adhesion peptides have been used in combination with rBM products previously for 3D hydrogel systems (*14*, *58*, *63*–*65*). To assess whether combinations of peptides could enhance acinar morphogenesis, we combined different types of peptide-modified alginates to present multiple ligands to the cells. Specifically, our experimental conditions comprised equal parts of IKVAV and RGD (1:1) and equal parts of YIGSR and RGD (1:1) tested under all mechanical conditions (fig. S7, A and B). In addition, we used IKVAV and RGD at a 3:1 ratio and equal parts of IKVAV, YIGSR, and RGD (1:1:1), both under the soft, fast-relaxing condition (fig. S7C).

Both soft, fast-relaxing, IKVAV and RGD (1:1) eBMs and soft, fast-relaxing, IKVAV and RGD (3:1) eBMs promoted round, polarized structures that were not significantly different in roundness or area from those clusters grown in soft, fast-relaxing, IKVAV-modified eBMs (fig. S7, D and E). We found that soft, fast-relaxing, IKVAV, YIGSR, and RGD (1:1:1) eBMs and soft, fast-relaxing, 3:1 (I:R) eBMs had no significant differences in roundness or area from the IKVAV-alone group (fig. S7, D and E). These groups also had basally polarized β4 integrin and laminin-5, with apically polarized GM130, which matches what we see in IKVAV-alone eBMs (fig. S7C). Expectedly, the soft, fast-relaxing, YIGSR- and RGD-modified eBMs produced clusters with a significantly lower roundness value than those clusters grown in soft, fast-relaxing, IKVAV-modified eBMs. Thus, while IKVAV can promote robust acini, RGD addition does not enhance this morphogenesis.

### MCF7 cells exhibit similar trends to those of MCF10A cells in modified eBMs

To evaluate the generalizability of the eBM with another cell line, we encapsulated cells from a tumorigenic but nonmetastatic breast cancer cell line (MCF7) (*66*, *67*). We found that soft, fast-relaxing, IKVAV-modified eBMs and soft, slow-relaxing, IKVAV-modified eBMs all promoted a rounded, noninvasive morphology (fig. S8A). In contrast, MCF7 cells in stiff, slow-relaxing, IKVAV-modified eBMs developed a grape-like cluster morphology (fig. S8A) (*68*). Similar to MCF10A cells, MCF7 cells in stiff, slow-relaxing, IKVAV-modified eBMs had lower cluster roundness and higher cluster area than those clusters in all other IKVAV-modified eBMs (fig. S8C). MCF7 cells also mimicked the phenotype we saw with MCF10A cells when encapsulated in RGD-modified eBMs. Irrespective of mechanical conditions, clusters took on an aberrant phenotype with a lack of polarization (fig. S8, B to D).

### Differential β4 and β1 integrin localization correlates with IKVAV and RGD peptides

After revealing notable differences in phenotype between MCF10A clusters in IKVAV- and RGD-modified eBMs, we were compelled to investigate the molecular mechanisms underlying the variation in phenotypes across these modified eBMs. We hypothesized that ligand-specific integrin signaling underlies the divergent phenotypes observed between IKVAV- and RGD-modified eBMs, as these motifs are bound by distinct integrin subunit combinations. IKVAV motifs are predominantly bound by β1 integrins (e.g., α3β1, α2β1, α6β1), while RGD motifs are engaged by αv and β1 integrins in MECs (*69*). It is important to note that while β1 integrins are crucial for IKVAV and RGD binding, other integrins also play important roles in MECs. For example, α6β4 integrin is a key receptor for laminin, a major component of the basement membrane, and is essential for hemidesmosome formation and cell adhesion (*70*).

**Fig. 5.**
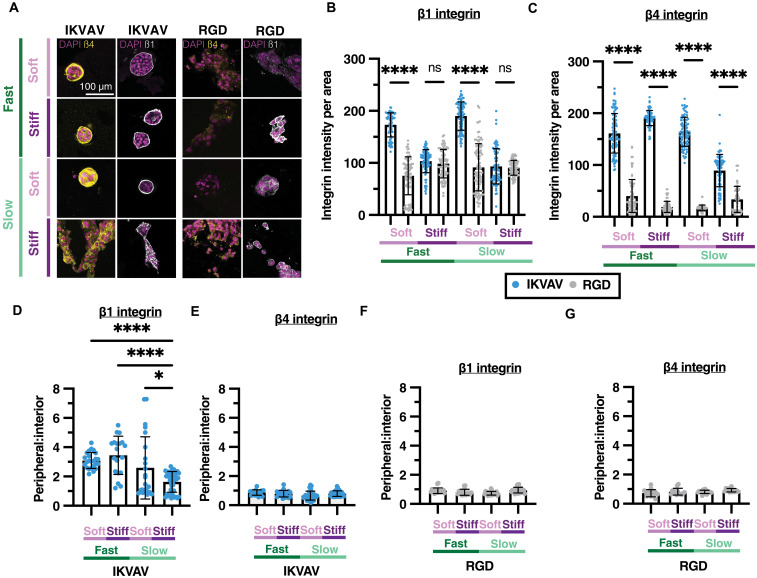
Polarization of β1 and β4 integrins associated with acinar development. (**A**) Representative confocal images of MCF10A cells encapsulated in high-concentration IKVAV–modified eBMs (left) and high-concentration RGD–modified eBMs (right)*.* From left to right: DAPI/β4 integrin and DAPI/β1 integrin. (**B**) Quantification of β1 integrin intensity between IKVAV- and RGD-modified eBMs. (**C**) Quantification of β4 integrin intensity between IKVAV- and RGD-modified eBMs. (**D**) Quantification of peripheral to inner β1 integrin within IKVAV-modified eBMs. (**E**) Quantification of peripheral to inner β4 integrin within IKVAV-modified eBMs. (**F**) Quantification of peripheral to inner β1 integrin within RGD-modified eBMs. (**G**) Quantification of peripheral to inner β1 and β4 integrins within RGD-modified eBMs. All scale bars, 100 μm. Data are shown as the means ± SD of three biological replicates (*n* = 3, 20 to 50 images per replicate), unless otherwise indicated. Intensity measurements show all individual measurements. Peripheral: Interior measurements were taken from *n* > 30 images total over three biological replicates. Statistical significance was determined by a one-way ANOVA and post hoc multiple comparison tests for integrin intensity and peripheral:interior ratio. *P < 0.05 and ****P < 0.0001.

In IKVAV-modified eBMs, β1 and β4 integrins were basally localized in fast-relaxing matrices (both soft and stiff) as well as in soft, slow-relaxing matrices (Fig. 5A). However, in stiff, slow-relaxing, IKVAV-modified eBMs, β1 and β4 integrins were more randomly distributed, with higher variability in signal intensity (Fig. 5A). Expectedly, we also saw dispersed organization of both β1 and β4 integrins under all RGD conditions (Fig. 5A). Furthermore, β1 integrins were significantly more abundant than β4 integrins in RGD-modified eBMs (fig. S9B).

To determine the relative abundance of β1 and β4 integrins, we quantified the mean intensity normalized by cell cluster area from confocal microscopy images. We saw a significantly higher β1 integrin intensity in soft, fast-relaxing, IKVAV-modified eBMs and soft, slow-relaxing, IKVAV-modified eBMs compared to their RGD-modified counterparts (Fig. 5B). We did not observe a significant difference in β1 integrin intensity in stiff, IKVAV-modified eBMs compared to their RGD-modified counterparts (Fig. 5B). Conversely, β4 integrin intensity was significantly higher in all IKVAV-modified eBMs compared to their RGD-modified eBM counterparts. Along with this, stiff, slow-relaxing, IKVAV-modified eBMs, which were the only group to induce malignant phenotypes, had the lowest β4 integrin intensity when compared to other IKVAV-modified eBMs (Fig. 5C and fig. S9A).

To compare the extent of polarization of each integrin subunit, we quantified the relative localization of peripheral versus interior integrin intensity. Notably, there was greater peripheral localization of β1 integrins in comparison to β4 integrins within IKVAV-modified eBMs (Fig. 5, A, D, and E). In both soft, fast-relaxing IKVAV-modified eBMs and stiff, slow-relaxing, IKVAV-modified eBMs, β1 integrin was most abundant on the cluster periphery, while β4 integrin was found at both the periphery and interior of the cluster (Fig. 5, D and E). However, there was a significant decrease in peripheral:interior β1 integrin in stiff, slow-relaxing, IKVAV-modified eBMs, where invasion was prominent (Fig. 5D). In contrast, there were no significant differences in the peripheral:interior localization of β1 and β4 integrins in slow-relaxing, RGD-modified eBMs (Fig. 5, F and G). On the basis of strong localization of both β1 and β4 integrins to the basal side of acini in IKVAV-modified eBMs, we hypothesized that there is a balance in activity between β1 and β4 integrins in this context. To explore this further, we investigated the downstream signaling events associated with β1 and β4 integrin activation in IKVAV- and RGD-modified eBMs.

### Hemidesmosome formation is promoted in soft, fast-relaxing, IKVAV-modified matrices

**Fig. 6.**
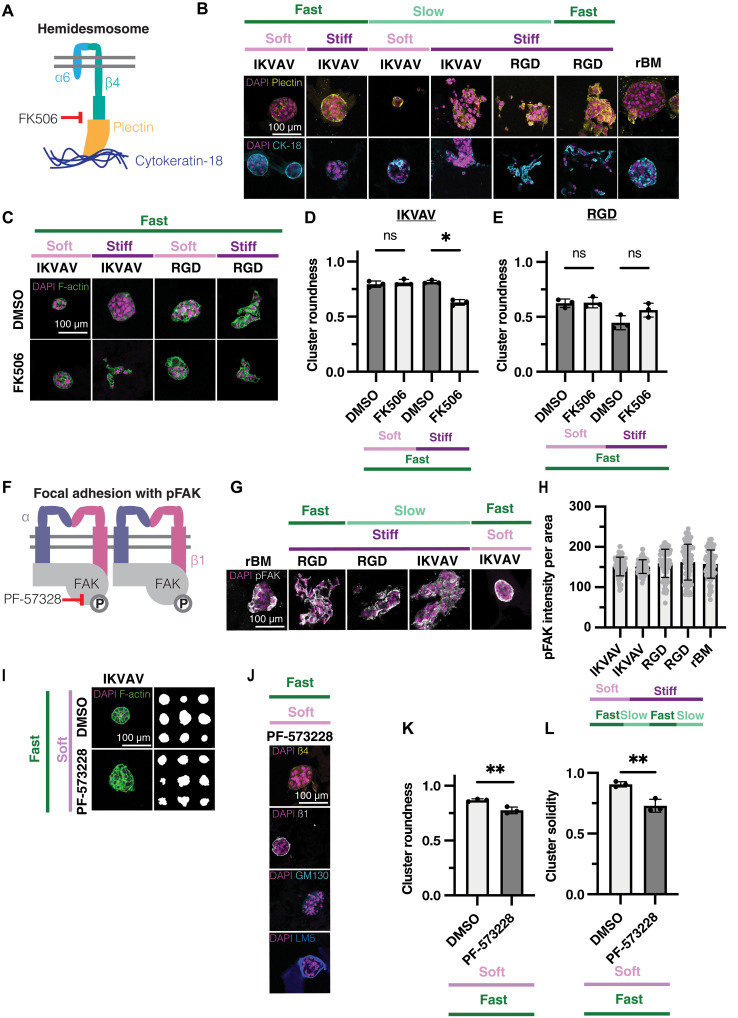
Both β1 and β4 integrin–based adhesion signaling leads to acinar formation in eBMs. (**A**) Schematic of hemidesmosome structures and inhibition with FK506. (**B**) Representative confocal images of MCF10A cells encapsulated in IKVAV- and RGD-modified eBMs and the rBM. DAPI and cytokeratin-18 (top) and plectin (bottom). (**C**) Representative confocal images of DMSO (control)– or FK506-treated MCF10A cells encapsulated in IKVAV- and RGD-modified eBMs. (**D**) Quantification for IKVAV-modified eBMs from (C). (**E**) Quantification for RGD-modified eBMs from (C). (**F**) Schematic of focal adhesions, FAK phosphorylation, and inhibition with PF-573228. (**G**) Representative confocal images of MCF10A cells encapsulated in IKVAV- and RGD-modified eBMs and the rBM. DAPI/pFAK. (**H**) Quantification of (G). (**I**) Representative confocal images of DMSO (control)– or PF-573228–treated MCF10A cells encapsulated in IKVAV-modified eBMs. DAPI/F-actin and representative outlines. (**J**) Representative confocal images of PF-573228–treated MCF10A cells encapsulated in IKVAV-modified eBMs. DAPI/β4 integrin, β1 integrin, GM130, and LM-332. (**K**) Quantification of cluster roundness from (I). (**L**) Quantification of cluster solidity from (I). All scale bars, 100 μm. Data are shown as the means ± SD of three biological replicates (*n* = 3, 20 to 50 images per replicate), unless otherwise indicated. Intensity measurements show all individual measurements. Statistical significance was tested by a Kruskal-Wallis test followed by Dunnett’s multiple testing correction for cluster roundness, by a one-way ANOVA and post hoc multiple comparison tests for solidity and pFAK intensity, and by a *t* test for cluster solidity (*P* value <0.05). *P < 0.05 and **P < 0.01.

Laminin-332 (LM-332)–bound α6β4 integrins are known to cluster at the cell membrane, enabling MECs to form hemidesmosomes that provide stable attachment to the ECM (*71*). The adaptor protein plectin connects the cytoplasmic tail of clustered β4 integrins to the keratin intermediate filament network (Fig. 6A, left). Previous research has identified that hemidesmosome-dependent polarity, along with ECM composition, correlates with the growth rate and acinus formation of MECs (*14*, *72*).

We found that plectin and cytokeratin-18 localized to the basal side of acini in the soft and stiff, fast-relaxing, IKVAV-modified eBMs, as well as soft, slow-relaxing, IKVAV-modified eBMs, similar to the rBM controls (Fig. 6B). The presence of clustered β4 integrins, plectin, and cytokeratin-18 indicates that hemidesmosome formation is likely a key contributor to the acinar phenotype in the fast-relaxing, IKVAV-modified eBMs and soft, slow-relaxing, IKVAV-modified eBMs. In stiff, slow-relaxing, IKVAV-modified eBMs and RGD-modified eBMs, which all yield malignant phenotypes, we did not see the basal localization of cytokeratin-18 and plectin. Instead, there was diffuse distribution of these proteins throughout the cell clusters (Fig. 6B).

To investigate the role of hemidesmosomes in maintaining the acinar phenotype, we inhibited their formation in MCF10A cells cultured in modified eBMs. FK506, a calcineurin inhibitor, disrupts hemidesmosomes by phosphorylating the β4 integrin cytoplasmic tail (Fig. 6A) (*73*), freeing α6β4 to activate proinvasive phosphatidylinositol 3-kinase (PI3K), Akt, and mitogen-activated protein kinase signaling (*74*). In soft, fast-relaxing, IKVAV-modified eBMs, FK506 treatment had no significant effect on cluster morphology (Fig. 6, C and D), suggesting that a soft, fast-relaxing, IKVAV-modified matrix does not promote invasion regardless of hemidesmosome status. However, in stiff, fast-relaxing, IKVAV-modified eBMs, inhibition of hemidesmosomes led to a marked decrease in roundness and the emergence of an invasive phenotype (Fig. 6, C and D), indicating that hemidesmosomes play a critical role in restricting invasion even in a stiff environment. Notably, RGD-modified eBMs showed no significant changes in phenotype or cluster roundness upon FK506 treatment (Fig. 6, C and E), demonstrating that this effect is specific to β4 integrin interactions in the IKVAV-modified matrix. Together, these findings suggest that hemidesmosomes help preserve the acinar phenotype and prevent invasion despite elevated matrix stiffness, while their loss enables invasive behavior.

### Phosphorylated FAK contributes to acinar morphogenesis in IKVAV-modified eBMs

To examine β1 integrin signaling, we assessed phosphorylated focal adhesion kinase (FAK) (pY397) in modified eBMs. β1 integrin activation leads to clustering at the membrane (*75*, *76*), recruitment and phosphorylation of FAK at Y397 (*77*–*79*), and ultimately, regulation of MEC responses to physical cues (Fig. 6F) (*80*, *81*). Unexpectedly, we observed strong pFAK intensity across all eBM conditions regardless of stiffness or stress relaxation (Fig. 6, G and H), with RGD-modified eBMs having more pFAK intensity overall compared to IKVAV-modified eBMs. Of note, the pFAK intensity from RGD- and IKVAV-modified eBMs was not significantly different from the pFAK intensity measured in the rBM (Fig. 6H). This finding was unexpected, as the phosphorylation of FAK has predominantly been associated with stiff matrices (*17*, *80*, *82*). FAK inhibition led to a significant decrease in cluster roundness in soft, fast-relaxing, IKVAV-modified eBMs, indicative of a disruption in acinus formation (Fig. 6, I, K, and L). We also found that FAK inhibition caused significant changes in the surface roughness of the cell clusters, quantified by solidity (Fig. 6L). Moreover, we also saw decreased cluster polarization of basal β4 integrin and apical GM130 (Fig. 6L). These findings reveal a role for FAK signaling in acinar morphogenesis, demonstrating that FAK signaling is crucial not only in stiffer matrices but also in softer environments.

### IKVAV-modified eBMs promote significant endogenous laminin deposition throughout the culture period

Inspired by a report that cells in engineered hydrogels can deposit matrix proteins dependent on the initial cross-linking density of the hydrogel (*83*), we investigated whether MCF10A clusters in modified eBMs secrete basement membrane proteins, potentially aiding acinus formation under specific conditions. As laminin is the most abundant protein of the basement membrane, we stained clusters in the modified eBMs with pan-laminin and LM-332 antibodies. Given that IKVAV is derived from laminin-111 (*48*, *53*, *84*), LM-332 should reflect only the laminin produced endogenously by cells.

**Fig. 7.**
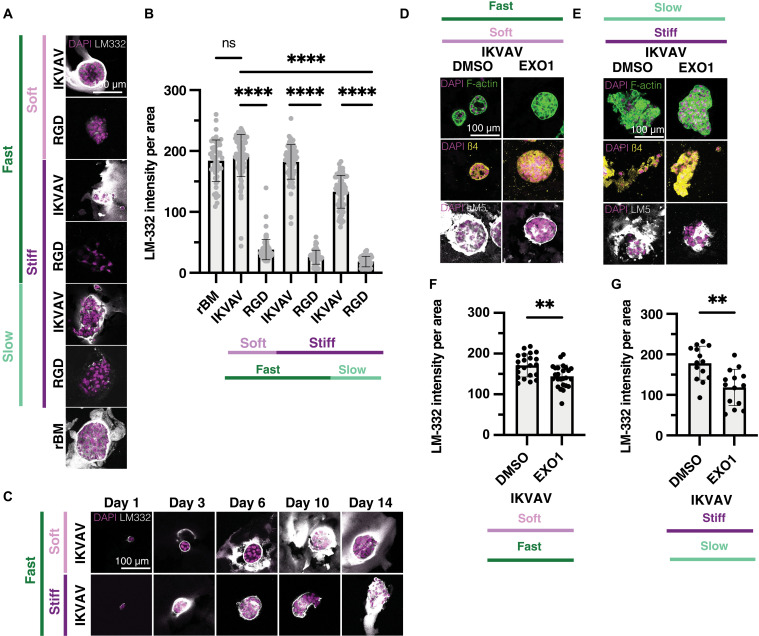
Only IKVAV eBMs support significant endogenous LM-332 deposition. (**A**) Representative confocal images of MCF10A cells encapsulated in IKVAV- and RGD-modified eBMs, as well as the rBM. DAPI/LM-332. (**B**) Quantification of LM-332 intensity from (A). (**C**) Representative confocal images of MCF10A cells encapsulated in soft, fast-relaxing and stiff, fast-relaxing, IKVAV-modified eBMs. Images taken at days 1, 3, 6, 10, and 14. DAPI/LM-332. All scale bars, 100 μm. (**D**) Representative images of MCF10A cells treated with DMSO (control) or Exo-1 in soft, fast-relaxing, IKVAV-modified eBMs. From top to bottom: DAPI/F-actin, DAPI/β4 integrin, and DAPI/LM-332. (**E**) Representative images of MCF10A cells treated with DMSO (control) or Exo-1 in stiff, slow-relaxing, IKVAV-modified eBMs. From top to bottom: DAPI/F-actin, DAPI/β4 integrin, and DAPI/LM-332. (**F**) Quantification of mean LM-332 intensity per area for soft, fast-relaxing, IKVAV-modified eBMs. (**G**) Quantification of mean LM-332 intensity per area for soft, fast-relaxing, IKVAV-modified eBMs. Data are shown as the means ± SD of three biological replicates (*n* = 3, 20 to 50 images per replicate), unless otherwise indicated. Intensity measurements show all individual measurements. Statistical significance was tested by a one-way ANOVA and post hoc multiple comparison tests for LM-332 intensity. **P < 0.01 and ****P < 0.0001.

Notably, LM-332 deposition was significantly increased in IKVAV-modified eBMs compared to RGD-modified eBMs (Fig. 7, A and B). In IKVAV-modified eBMs, laminin was predominantly localized adjacent to the basal side of the cell clusters (Fig. 7A), with soft, fast-relaxing, IKVAV-modified eBMs exhibiting significantly higher laminin deposition than their stiff, slow-relaxing counterparts (Fig. 7B). In contrast, laminin deposition in RGD-modified eBMs was sparse and primarily confined within the cell clusters rather than surrounding them (Fig. 7A), and the overall levels were markedly lower than those observed in IKVAV-modified eBMs (Fig. 7B). We observed similar trends when staining with a pan-laminin antibody (fig. S10, A and B). Of note, we see less LM-332 deposition in MCF7 cells compared to MCF10A cells, although LM-332 is basally polarized in acinar-like structures (fig. S8A). This difference may occur because MCF7 cells are a luminal cell type and may produce less matrix (*66*, *85*).

Next, we investigated the timescale of laminin deposition by immunostaining and imaging IKVAV-modified eBMs at intermediate time points within the culture period. Laminin was deposited as early as day 1 and was continuously produced throughout the 14-day experimental time course (Fig. 7C). LM-332 deposition between soft and stiff, fast-relaxing, IKVAV-modified eBMs was similar by the end of the time points (Fig. 7C). The strong LM-332 deposition in IKVAV-modified eBMs, but not in RGD-modified eBMs, suggests that MCF10A clusters in modified eBMs not only respond to their environment but also contribute to endogenous LM-332 deposition, potentially enhancing cellular polarization and functionality over time, depending on the condition. Furthermore, this outcome underscores the critical role of the initial eBM condition provided to the cell, highlighting its influence on both the quantity and nature of the endogenous proteins synthesized.

We next sought to further investigate the link between acinus development and nascent protein deposition in IKVAV-modified eBMs. Researchers have used Exo-1, an inhibitor of the secretory pathway, to diminish nascent protein production in engineered hydrogels (*86*, *87*). Exo-1 treatment in soft, fast-relaxing, IKVAV-modified as well as stiff, slow-relaxing, IKVAV-modified eBMs leads to a significant decrease in LM-332 production, yet acini still developed in these matrices with no observable differences in phenotype compared to the dimethyl sulfoxide (DMSO)–treated controls (Fig. 7, D to G). Collectively, these results indicate that the IKVAV motif alone is sufficient to promote mammary acinar morphogenesis, provided that the matrix exhibits an appropriate combination of mechanical properties. Furthermore, a balanced engagement of β1 and β4 integrins appears to be crucial for successful acinar development in IKVAV-modified eBMs.

## DISCUSSION

We have designed a tunable 3D matrix system with definable parameters for culturing MECs that enables mammary acinar morphogenesis without the rBM. This system allows for the independent manipulation of both biochemical and mechanical properties, effectively decoupling their effects, which has been a challenge with traditional rBM systems. Soft, fast-relaxing, IKVAV-modified eBMs, designed to mimic the rBM, promoted polarized acini in MECs.

We also observed an acinar-like phenotype in both the stiff, fast-relaxing, IKVAV-modified eBMs and soft, slow-relaxing, IKVAV-modified eBMs. This optimized platform overcomes limitations of existing synthetic systems, which have either required exogenous matrix components or lacked the independent control over matrix mechanics necessary for complete acinar polarization. Self-assembling peptide hydrogels like PeptiGel have been explored for rBM-free mammary epithelial cell culture, but achieving complete acinar maturation has required exogenous laminin supplementation (*88*). In addition, while polyethylene glycol hydrogels have shown promise for mammary acinus formation through stiffness optimization and RGD peptide conjugation, they still lack independently tunable viscoelasticity (*89*). Moreover, our work demonstrates the substantial positive impact of IKVAV adhesion motifs on acinar morphogenesis in comparison to RGD motifs. We have also successfully cultured mammary murine and patient-derived tumor organoids in IKVAV-modified eBMs, demonstrating that modified eBMs can be versatile and applicable to other biological systems.

Prior reports have elegantly shown in rBM-based systems that matrix stiffness in the range of malignant breast tumors (1 to 5 kPa) drives invasion in MECs (*14*, *15*, *82*). However, in our IKVAV-modified eBMs, tumor-like stiffness only induced invasion when the matrix was both slow relaxing and stiff. Several factors may explain this difference. First, the stress relaxation rates of the eBM play a crucial role in integrin clustering. Fast-relaxing matrices facilitate integrin clustering more effectively compared to slow-relaxing matrices (*90*, *91*). Second, we are independently modulating the stress relaxation properties of the eBMs, a mechanical perturbation that has not been previously characterized in clusters grown from single MECs in rBM-free systems. Last, while the use of an rBM-free system allows us to unravel the contributions of different ECM components in a reductionist manner, there will be inherent differences from experiments performed with the complexity of rBM components. These could include the presence of growth factors and other signaling molecules from rBM products, which have been reported to affect cell culture studies as well as contributions from the complex interactions of basement membrane structural proteins that are not recapitulated by peptide-modified alginate networks (*32*–*34*).

Although IKVAV and YIGSR are both derived from laminin-111, there are apparent differences in their ability to promote MCF10A acinar morphogenesis in our eBM hydrogel platform. There are a few mechanisms that may explain differences in phenotype these ligands promote in our modified eBMs. For one, IKVAV and YIGSR are located on the α and β chains of laminin-111, respectively (*48*, *92*). In addition, while these peptides bind to the same set of integrins, they may exhibit different binding affinities (*92*). Moreover, YIGSR is also able to bind to 67LR, a cell surface receptor for laminin (*48*), which may limit its ability to be bound by integrin molecules.

An invasive phenotype was present in RGD-modified eBMs regardless of their mechanical properties. The difference in phenotypes between the IKVAV- and RGD-modified eBMs clearly demonstrates the substantial impact of the chosen peptide motif on acinar development. Different integrin subunit combinations bind the IKVAV and RGD motifs and thus induce different integrins to cluster, subsequently forming distinct adhesion complexes, leading to the emergence of the observed phenotypes. In MECs, β1 and β4 integrins play a role in both polarized and invasive phenotypes, with the ECM modulating their functions (*93*). For instance, β4 integrins promote invasion via the PI3K pathway (*94*) but are also involved in hemidesmosome formation and acinar development. The role of β1 integrins is more complex; blocking β1 can reverse malignant phenotypes (*15*), while α2β1 integrins activate Rac and promote epithelial polarization (*95*). Notably, α2β1 is enriched in healthy mammary tissue but down-regulated in tumors (*96*, *97*).

We found that β4 integrin consistently localized to the basal side of acinar-like clusters. However, in invasive clusters, like in the stiff, slow-relaxing, IKVAV-modified eBMs, β4 integrin was randomly dispersed. We anticipate that differences in β4 integrin localization may explain the phenotypic differences we observed. We expect that cells may cluster integrins more effectively in fast-relaxing matrices (*39*) because of the physical remodeling of the alginate network, thus likely facilitating integrin polarization and hemidesmosome formation. Building on this, the observed lack of acinar morphogenesis in alginate gels with low IKVAV concentration could result from insufficient integrin-ligand complexes that prevent the necessary clustering for downstream signaling to initiate downstream signaling. Inhibition of both FAK and hemidesmosome formation disrupted acinar development in IKVAV-modified eBMs. Given that FAK is linked to β1 integrin signaling and hemidesmosomes to β4 integrin signaling, these results demonstrated that both β1 and β4 integrin signaling is critical for the acinar phenotype in these IKVAV-modified eBMs. Specifically, β4 integrin’s role in hemidesmosome formation is required for acinar development in stiff, fast-relaxing, IKVAV-modified eBMs. Prior work has shown that enhanced stiffness can drive the invasive phenotype via PI3K and Rac1 signaling from the tail of β4 integrin if matrix mechanical conditions prevent hemidesmosome formation (*14*, *70*, *98*, *99*). Our results similarly show that matrices hinder β4 integrin polarization, such as stiff and invasive clusters. Fast stress relaxation, even in stiff matrices, enables hemidesmosome formation and prevents invasion. In addition, phosphorylated FAK staining, downstream of β1 integrin activity, revealed strong signal intensity in the acini formed in soft, fast-relaxing, IKVAV-modified eBMs. Inhibition of FAK signaling resulted in a significant reduction in cluster roundness and a disruption of cluster organization. Our findings suggest that the acinar phenotype in IKVAV-modified eBMs arises from a combination of mechanical and cell adhesion factors that promote a balance of β1 and β4 integrin activity.

This study observed increased endogenous laminin deposition in soft, fast-relaxing, IKVAV-modified eBMs compared to their stiff, slow-relaxing counterparts, which highlights the role of matrix compliance in facilitating ECM protein deposition. A more compliant, fast-relaxing matrix may alleviate physical constraints, thereby enhancing laminin deposition, consistent with previous findings (*83*, *100*). Furthermore, the observed laminin deposition in both stiff and soft, fast-relaxing, IKVAV-modified eBMs, contrasted with its absence in RGD-modified eBMs, underscores the critical influence of the adhesion peptide motif on matrix deposition and cellular phenotype. Elevated laminin levels are likely to drive acinus formation through multiple mechanisms, including the promotion of hemidesmosome formation through enhanced integrin clustering and protection of the nucleus from mechanical deformation (*14*, *101*). Consistent with this, hemidesmosomes were evident in IKVAV-modified eBMs, while the inhibition of their formation resulted in an invasive phenotype exclusively in stiff, fast-relaxing, IKVAV-modified eBMs. Notably, no phenotypic differences were observed in the RGD-modified eBM control or treated groups, further highlighting the unique role of IKVAV in promoting acinar-like organization. In addition to hemidesmosome formation, laminin deposition may contribute to nuclear stability. Recent studies suggest that high laminin levels form a keratin-laminin shield, protecting the nucleus from mechanical deformation and dampening cellular responses to mechanical cues (*101*). This protective mechanism may explain how increased laminin deposition in stiff, fast-relaxing, IKVAV-modified eBMs supports nuclear integrity, ultimately favoring acinar morphology. Last, a cascading chain of events leads to α6β4 integrin clustering: α2β1 integrin activation drives LM-332 deposition and organization, which in turn create additional binding sites, culminating in α6β4 integrin clustering (*72*, *95*, *102*). This integrin-mediated positive feedback loop likely plays a pivotal role in reinforcing acinar phenotypes in IKVAV-modified eBMs (*79*). Together, these findings emphasize the interplay between matrix mechanics, laminin deposition, and integrin signaling in driving acinus formation.

While this study focused on IKVAV-, YIGSR-, and RGD-modified eBMs, additional adhesion motifs could be conjugated to an alginate matrix to mimic more components of rBM, for example, collagen IV, which constitutes ~30% of the basement membrane (*33*). This approach would refine our understanding of the specific peptides required for mammary morphogenesis. Furthermore, combining collagen and laminin adhesion motifs would present more representative basement membrane signals to cells, enabling more complex studies of ECM ligand interactions and their effects on mammary epithelial cell phenotype.

Although our reductionist matrix allows precise control over individual mechanical and biochemical cues—offering a powerful tool to dissect their distinct roles—it does not replicate the full complexity of rBM, which contains more than 2000 proteins that may contribute synergistically to certain biological processes (*9*, *33*). While this complexity poses challenges for reproducibility and mechanistic studies, it also presents utility in applications where an undefined, yet functionally rich microenvironment is beneficial. Our approach, therefore, complements existing models by enabling controlled investigations into ECM properties that would be difficult to isolate in traditional rBM-based systems. In addition, this platform has the potential to be extended to other cell types requiring precise modulation of multiple mechanical and biochemical cues to understand their individual contributions.

Beyond mammary epithelial morphogenesis, our eBM system offers broad utility for studying mechanotransduction, ECM cues, and integrin signaling across diverse biological contexts. The modularity of this platform enables the incorporation of additional ECM-derived motifs to further refine basement membrane mimicry, paving the way for more physiologically relevant in vitro models. Moreover, the successful culture of patient-derived tumor organoids in our system underscores its potential for translational applications, including precision medicine and therapeutic screening. By bridging the gap between reductionist synthetic matrices and the complexity of native basement membranes, this platform provides a powerful tool to unravel fundamental mechanisms governing tissue organization, disease progression, and cellular responses to the microenvironment.

## MATERIALS AND METHODS

### Materials

Purified Pronova UPVLVG and Pronova LF20/40 alginates were purchased from Novamatrix. RGD, YIGSR, and IKVAV peptides were obtained from Peptide 2.0. Full peptide sequences and their molecular weight are as follows: GGGGRGDSP (758.75 g/mol), CQAASIKVAV (989.19 g/mol), and CDPGYIGSR (967.06 g/mol). Growth factor–reduced Matrigel was purchased from Corning (cat. no. 354230).

### Alginate preparation, conjugation, and characterization

Pronova LF20/40 alginate was purified by dialysis (3500–molecular weight cutoff tubing) against Milli-Q water for 4 days, frozen, and lyophilized until it dried. LF20/40 (high molecular weight) or VLVG (low molecular weight) alginate was dissolved in 0.1 M MES buffer overnight. Carbodiimide chemical reactions were carried out in a 0.1 M MES buffer (pH 6.5) solution to modify alginate chains with their respective peptides. Peptides (either RGD, YIGSR, or IKVAV) were conjugated to the alginate backbone via primary amines. Separate batches of alginate were made for each respective peptide (RGD, YIGSR, or IKVAV). 1-Ethyl-(dimethylaminopropyl) carbodiimide (EDC) was used to form amide linkages between amine-containing molecules of the peptides and the carboxylate moieties on the alginate polymer backbone. *N*-Hydroxy-sulfosuccinimide was used as a co-reactant to stabilize the reactive EDC intermediates and enhance conjugation efficiency. *N*-Hydroxy-sulfosuccinimide, EDC, and respective peptides were added quickly and sequentially to an alginate solution to achieve homogeneously modified alginate. The reaction was carried out for 20 hours and then quenched with 100× hydroxylamine [alginate (125 mg/g)]. Activated charcoal was added to each batch of alginate and mixed for 30 min, allowing the charcoal to settle afterward. The alginate was then filtered twice, first through a 0.8-μm filter and then through a 0.2-μm filter. After filtration, the alginate was frozen at −80°C before lyophilization. The samples were lyophilized at −50°C for 72 hours. Once complete, the alginate was reconstituted under sterile conditions in Dulbecco’s modified Eagle medium (DMEM)/F12 basal media and used at a final concentration of 1% in the gels. The degree of peptide conjugation was approximated via ^1^H NMR spectroscopy (500 MHz, Bruker, Germany). Alginate was reconstituted in sterile D_2_O (deuterium oxide) at 0.1, 0.5, 1, or 2%. The ^1^H NMR spectra and corresponding peak assignments for each sample are provided in the Supplementary Materials (figs. S1 and S2 table S1).

### Rheology

Rheology experiments were conducted using a stress-controlled Anton Paar MCR 502 rheometer. Briefly, the gels were fabricated and deposited directly onto the bottom plate, and a 20-mm plate was then lowered slowly to contact the gel. Mineral oil was deposited to the periphery of the gel to avoid gel dehydration. A time sweep was conducted (1 Hz, 1% strain, 37°C), and the storage (*G*′) and loss moduli (*G*″) were recorded over time. From these data, the complex modulus (G*) was calculated (Eq. 1), and subsequently, the elastic modulus (*E*) was calculated (Eq. 2). Poisson’s ratio (*v*) was assumed to be 0.5. Once the storage modulus reached an equilibrium value, a stress relaxation test was subsequently performed at 10% strain. The stress relaxation time was defined as the time taken for the maximum stress to relax to half of its initial value.

$$G^{*}={\left({G^{\prime }}^{2}+G^{\prime \prime 2}\right)}^{1/2}$$ (1)

$$E=2\;\left(1+v\right){\text{ G}}^{*}$$ (2)

### Mammary epithelial cell culture

Immortalized, nontumorigenic MCF10A mammary epithelial cells were obtained from American Type Culture Collection (ATCC) and cultured via established protocols (*1*). Cells were cultured as a monolayer in 2D tissue culture flasks until the time for encapsulation. The following growth medium recipe was used: DMEM/F12 (Thermo Fisher Scientific) basal media were supplemented with 5% horse serum (Thermo Fisher Scientific), 1% penicillin/streptomycin (Thermo Fisher Scientific), epidermal growth factor (20 ng/ml; Peprotech), hydrocortisone (0.5 mg/ml; Sigma-Aldrich), cholera toxin (100 ng/ml; Sigma-Aldrich), and insulin (10 μg/ml; Sigma-Aldrich). Before encapsulation, MCF10A cells were washed with phosphate-buffered saline (PBS), trypsinized (0.05% trypsin/EDTA; Thermo Fisher Scientific), spun down [2000 rcf (relative centrifugal force), 5 min], counted, and resuspended in DMEM/F12 to create a single-cell suspension with a final concentration in the hydrogels of 50,000 cells/ml.

MCF7 cells were obtained from ATCC and cultured in media with DMEM [d-glucose (4.5 g/liter); Thermo Fisher Scientific], 10% fetal bovine serum (Thermo Fisher Scientific), and 1% penicillin/streptomycin (Thermo Fisher Scientific). Cells were cultured as a monolayer in 2D tissue culture flasks until the time for encapsulation. Before encapsulation, MCF10A cells were washed with PBS, trypsinized (0.25% trypsin/EDTA; Thermo Fisher Scientific), spun down (2000 rcf, 5 min), counted, and resuspended in DMEM to create a single-cell suspension with a final concentration in the hydrogels of 50,000 cells/ml.

### eBM formation

Alginate (1% w/v final concentration) in DMEM/F12 and a single-cell suspension (final concentration of 50,000 cells/ml) in growth media were mixed in a Luer lock syringe. For eBMs with multiple kinds of alginates, alginates were mixed at this stage in the syringe. Calcium sulfate was used to cross-link alginate matrices. A calcium sulfate slurry was mixed with DMEM/F12 and added to a second Luer lock syringe. Final calcium concentrations for soft and stiff matrices were 4 and 22 mM for LF20/40 matrices and 7 and 25 mM for VLVG matrices, respectively. The two syringes were connected via a coupler, and the solutions were mixed several times. The hydrogel solution was then quickly deposited into well plates of a 48-well plate (~500 μl per well). Hydrogels were incubated at 37°C for 2 hours before adding growth media and were then cultured for 2 weeks. Media were changed every 1 to 2 days.

### MCF10A cell encapsulation in the rBM

For MCF10A encapsulation into the rBM, Matrigel (Corning, cat. no. 354230) was mixed with a single-cell suspension (DMEM/F12), and gels were deposited into the wells of a 48-well plate (~500 μl per well). rBM matrices were incubated at 37°C for 2 hours before adding growth media and were then cultured for 2 weeks. Media were changed every 1 to 2 days.

### Murine mammary organoid culture

Murine mammy organoids were harvested in accordance with established protocols (*103*). In short, mammary glands from 8-week-old female mice (C57BL/6 strain) were minced in PBS, followed by digestion in a collagenase solution on a shaker. A 10-ml collagenase solution was prepared per mouse by combining 9 ml of DMEM/F12 (Thermo Fisher Scientific, no. 11320082), 0.5 ml of fetal bovine serum (Gibco), 5 μl of insulin (Sigma-Aldrich, cat. no. I-1882), 10 μl of gentamicin (50 mg/ml stock, Thermo Fisher Scientific, cat. no. 15750-485 060), 200 μl of collagenase (100 mg/ml stock, Sigma-Aldrich, no. 2139), and 200 μl of trypsin (100 mg/ml stock, Sigma-Aldrich, no. T7409). The fatty layer was isolated, washed with DMEM/F12, and combined with the original pellet. This combined material was treated with deoxyribonuclease and washed repeatedly, and then the organoids were encapsulated in IKVAV-modified eBMs and the rBM. These were maintained in DMEM/F12 media with penicillin/streptomycin and Insulin-Transferrin-Selenium (ITS). This research did not involve live animal work and thus did not require an animal use protocol. Mouse mammary gland tissue was acquired postmortem as a discarded by-product of mice euthanized under an unrelated Institutional Animal Care and Use Committee–approved protocol, and this tissue would have otherwise been disposed of.

### PDM-350 culture

HCM-CSHL-0261-C50 tumor organoids were obtained from ATCC and cared for via established ATCC protocols. Advanced DMEM/F12 was supplemented with Organoid Growth Kit 1F (ACS-7105), l-glutamine (ATCC, 30-2214), Hepes (Thermo Fisher Scientific, 15630080), B-27 Supplement (Thermo Fisher Scientific, 17504-044), and HA-R-Spondin1-Fc 293T (RSPO1) conditioned media (Trevigen, cat. no. 3710-001-01) for culture media. Organoids were thawed, centrifuged into a pellet, and resuspended in 100 to 200 μl of Matrigel for culture. Ten-microliter droplets or domes were dispensed in a six-well plate with about 10 droplets per well. The six-well plate was inverted and placed in a 37°C incubator for 30 min. After incubation, six-well plates were placed upright, and 2.0 ml of growth media was added per well. Media were changed every 1 to 2 days.

### PDM-350 encapsulation in eBMs and the rBM

Organoids were scraped from the six-well plate, centrifuged into a pellet, and resuspended in TrypLE (Thermo Fisher Scientific, no. 12604013) to generate single cells. The single cells were quenched, pelleted again, and resuspended in growth media or Matrigel for encapsulation.

### Inhibitor studies

All inhibitors were diluted in growth media and added 1 day postencapsulation. DMSO was used as a vehicle control for all inhibitors. FK506 (Cayman Chemical, 10007965) was used at a working concentration of 1 μM. PF-573228 (FAK inhibitor, Cayman Chemical) was used at a working concentration of 5 μM. Exo-1 (Sigma-Aldrich, 75541-83-2) was used at a working concentration of 120 μM.

### Immunofluorescence staining and confocal microscopy

Hydrogel samples were fixed in 4% paraformaldehyde in PBS for 45 min at 37°C. Samples were then washed twice with Dulbecco’s PBS with Ca^2+^/Mg^2+^ for 15 min, followed by an overnight incubation in a 30% (v/v) sucrose-cPBS solution to dehydrate the hydrogels. The hydrogels were then placed in a solution of equal volumes of optimal cutting temperature compound (Tissue-Tek) and 30% sucrose-cPBS for 4 to 6 hours. After incubation, the solution was removed, and the gels were placed individually into cryomolds with the optimal cutting temperature compound and then frozen at −20°C. The samples were then sectioned with a cryostat (Lecia CM1950, 40 μm) for immunostaining. Sections were washed with PBS and then incubated in blocking buffer [10% goat serum (Thermo Fisher Scientific), 1% bovine serum albumin (Sigma-Aldrich), 0.1% Triton X-100 (Sigma-Aldrich), and 0.3 M glycine (Sigma-Aldrich)] for 1 hour before the addition of primary antibodies. Samples were incubated with primary antibodies overnight and then rinsed with blocking buffer twice. Alexa Fluor 488–phalloidin (1:100 dilution; Thermo Fisher Scientific, A12379), 4′,6-diamidino-2-phenylindole (DAPI; 1 μg ml^−1^), and fluorescently conjugated secondary antibodies were diluted in the blocking buffer and added to slides for 1 hour at room temperature. Specific primary antibodies used were as follows: GM130 (1:100; BD Biosciences, 61083), β4 integrin (1:150; Life Sci. Tech., MA5-17104), β1 integrin (1:200; Thermo Fisher Scientific, 14-0299-82), plectin (1:100; Abcam, EPR26993-49), keratin-14 (1:234; Abcam, AB312312-1001), and LM-332 (1:100; P3H9, DSHB). Specific secondary antibodies used were as follows: Alexa Fluor IgG1 goat anti-mouse 488 (Thermo Fisher Scientific, A21121), Alexa Fluor IgG1 goat anti-mouse 555 (Thermo Fisher Scientific, A21127), and Alexa Fluor IgG2 goat anti-rabbit 488 (Thermo Fisher Scientific, A11008). Slides were imaged with a 25× objective on a Leica SP8 confocal laser scanning microscope.

### Image analysis

ImageJ [NIH (National Institutes of Health)] was used for image analysis to evaluate the roundness, area, and invasion of cell clusters, along with the localization of specific markers. At least three replicates (20 to 50 images per replicate) were used per condition, unless otherwise stated in the figure caption. Confocal images were thresholded and masked using ImageJ software to create outlines and analyze shape metrics of each cluster. Any missing “holes” in the image were then filled. Using this method, roundness or the inverse of the aspect ratio (4 × area/π × major_axis^2^) and area (μm^2^) were calculated. The average of each experimental replicate is displayed as one data point. Fluorescence intensity analysis involved calculating the mean intensity per unit area (μm^2^) for each cluster to facilitate comparisons across conditions. Interior-to-peripheral intensity measurements were performed by defining a 10-μm boundary surrounding the cell cluster to represent the “basal membrane” for peripheral intensity measurements. Interior intensity measurements were taken from the region within this boundary, excluding a 5-μm margin to avoid overlap with the peripheral zone. The percentage of invasive clusters was determined by classifying a cluster as invasive if it displayed a distinct protrusion, characterized by a discontinuous change in curvature at the resolution of the bright-field image (~1 μm).

### Statistical analysis

All data were analyzed in GraphPad Prism version 9.3.1 software using the tests described in the respective figure captions. *P* values of less than 0.05 were considered statistically significant. Unless otherwise noted, graphs represent three biological replicates per condition (*n* = 3), with each replicate representing the average at least 20 to 50 images.
