# Supplementary material for: Engineered basement membrane mimetic hydrogels to study mammary epithelial morphogenesis and invasion
Source: Sci Adv. 2025 Sep 26;11(39):eadx2110. doi: 10.1126/sciadv.adx2110 (PMC12466923; doi:10.1126/sciadv.adx2110)
Supplement: Supplementary file 1 — Figs. S1 to S10 Table S1 [file sciadv.adx2110_sm.pdf]

Science Advances
AAAS

Supplementary Materials for

Engineered basement membrane mimetic hydrogels to study mammary epithelial morphogenesis and invasion

Jane A. Baude *et al.*

Corresponding author: Ryan S. Stowers, rstowers@ucsb.edu

Sci. Adv. **11**, eadx2110 (2025)
DOI: 10.1126/sciadv.adx2110

This PDF file includes:

Figs. S1 to S10
Table S1

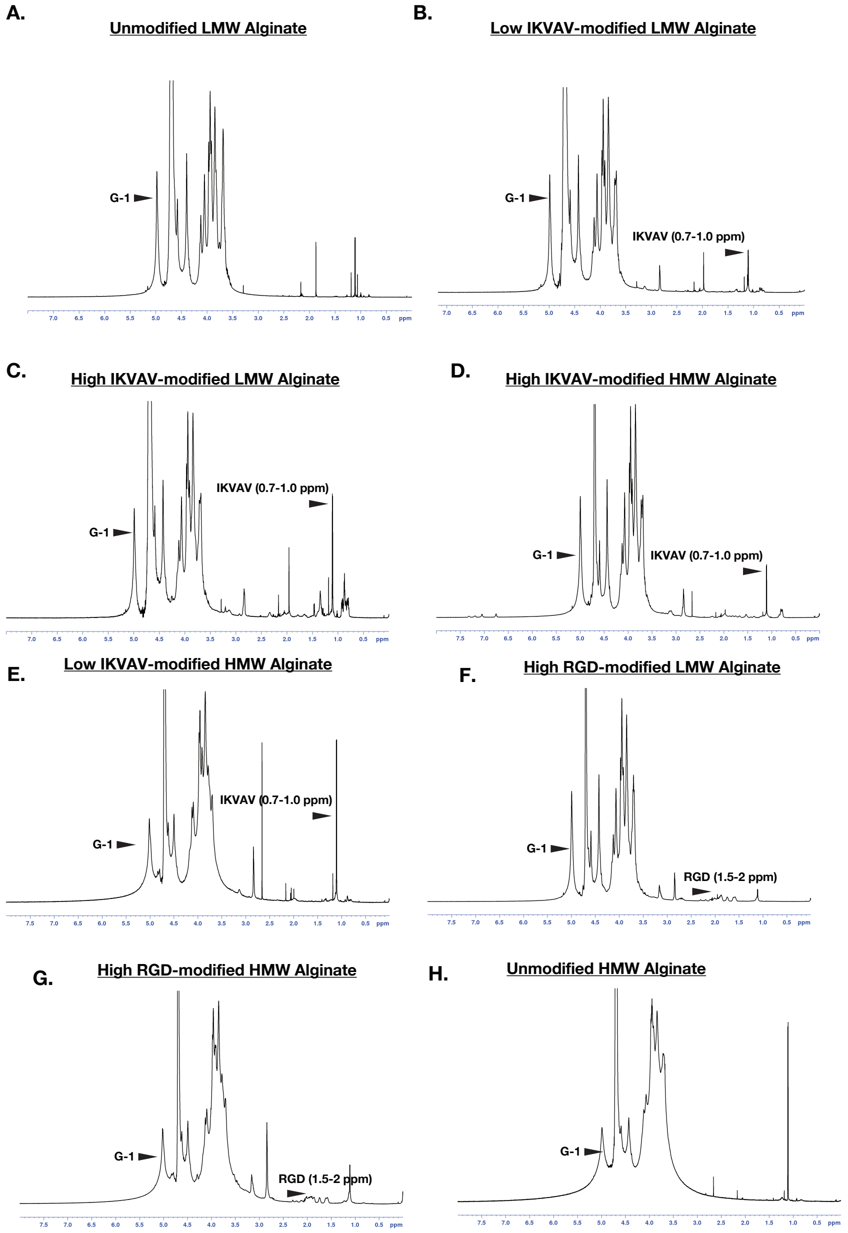

Fig. S1. ^{1}H-NMR spectra of unmodified alginate, IKVAV-modified and RGD-modified alginate. LMW = low molecular weight (fast-relaxing) and HMW = high molecular weight (slow-relaxing). (A) Unmodified fast-relaxing alginate. (B) Low concentration IKVAV-modified fast-relaxing alginate. (C) High concentration IKVAV-modified fast-relaxing alginate. (D) High concentration IKVAV-modified slow-relaxing alginate. (E) Low concentration IKVAV-modified slow-relaxing alginate. (F) High concentration RGD-modified fast-relaxing alginate. (G) High concentration RGD-modified slow-relaxing alginate. (H) Unmodified slow-relaxing alginate. One sample per condition was analyzed.

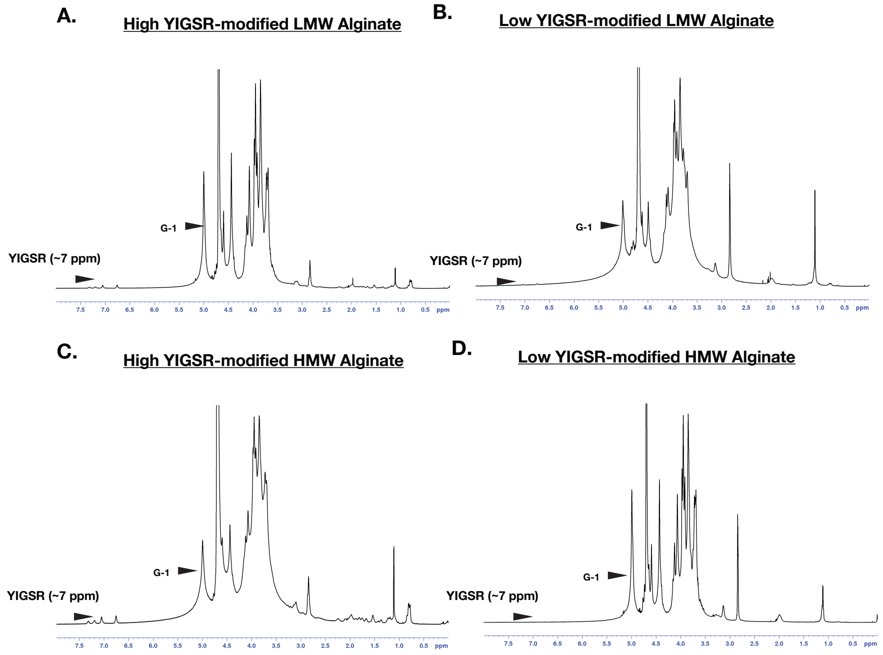

Fig. S2. 1H-NMR spectra of YIGSR-modified fast-relaxing and slow-relaxing alginate at varying concentrations. (A) High-concentration YIGSR-modified fast-relaxing alginate. (B) Low-concentration YIGSR-modified fast-relaxing alginate. (C) High-concentration YIGSR-modified slow-relaxing alginate. (D) Low-concentration YIGSR-modified slow-relaxing alginate. One sample per condition was analyzed.

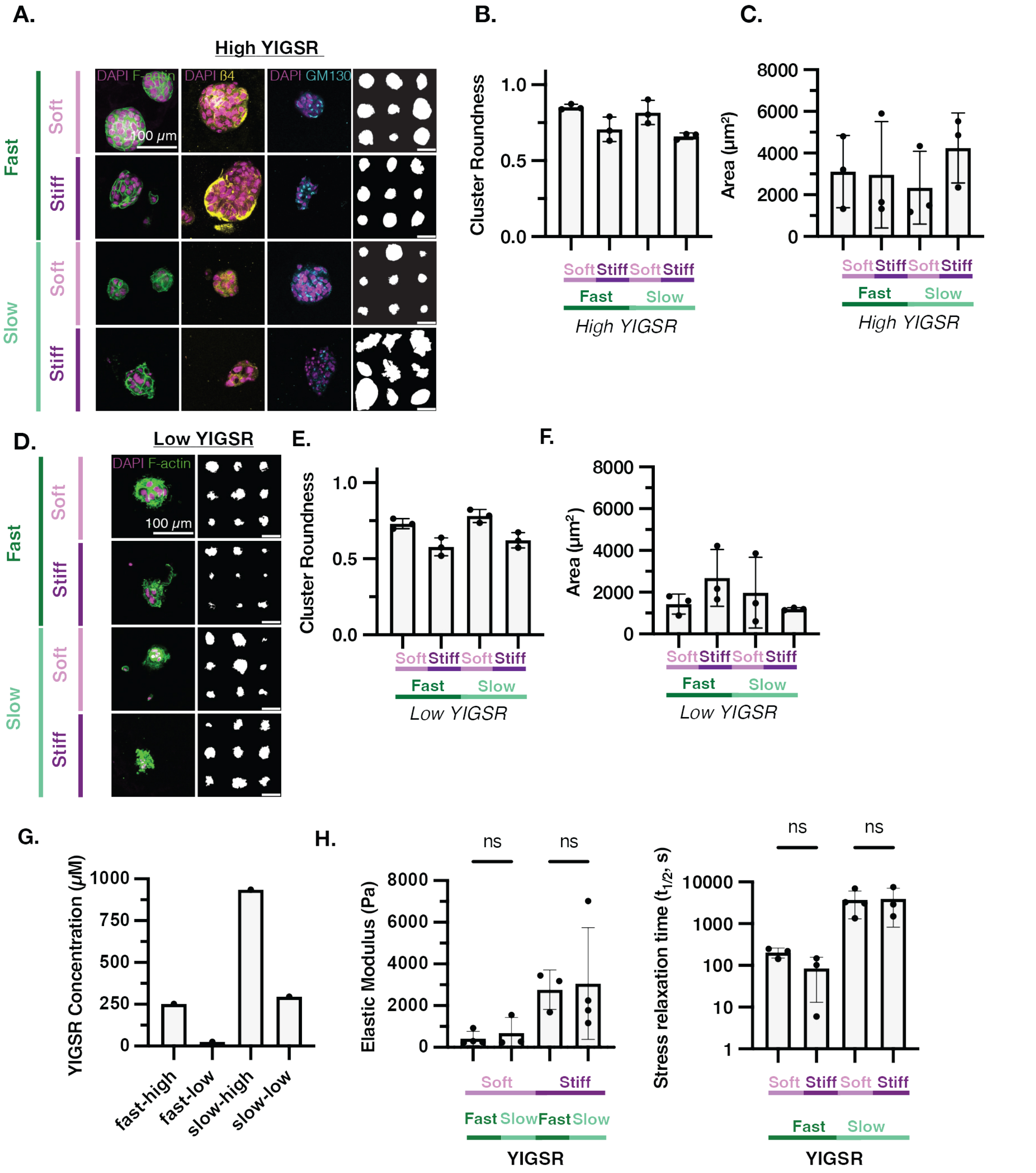

Fig. S3. YIGSR eBMs do not support robust mammary acinar morphogenesis. (A) Representative confocal images of MCF10As encapsulated in high concentration YIGSR-modified eBMs. From left to right: DAPI/F-actin, DAPI/β4-integrin, DAPI/GM130, and representative outlines of the respective condition. (B) Quantification of cluster roundness for high concentration YIGSR-modified eBMs. (C) Quantification of cluster area for YIGSR-modified eBMs. (D) Representative confocal images of MCF10As encapsulated in low concentration YIGSR-modified eBMs. DAPI/F-actin. Scale bars = 100 µm. (E) Quantification of cluster roundness for low concentration YIGSR-modified eBMs. (F) Quantification of cluster area for low concentration YIGSR-modified eBMs. (G) Summary of concentration of YIGSR peptide on modified fast- relaxing and slow-relaxing alginate from H-NMR data (Fig. S2, Table S1). (H) Elastic modulus of YIGSR-modified eBMs. (I) Stress relaxation rate of YIGSR-

modified eBMs. All scale bars = 100 µm. Data are shown as mean ± SD of three biological replicates ($n=3$, 20-50 images per replicate) unless otherwise indicated. Statistical significance was tested by a Kruskal-Wallis test followed by Dunnett's multiple testing correction for cluster roundness, and by one-way ANOVA and post hoc multiple comparison tests for cluster area. Significance was determined by one- way ANOVA for elastic modulus and stress relaxation time. If no statistical significance indicator bars are shown, there were no significant differences (p-value > 0.05). * indicates $p < 0.05$, ** $p < 0.01$, *** $p < 0.001$, **** $p < 0.0001$.

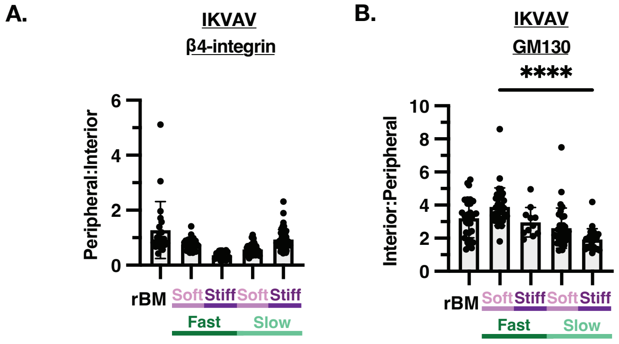

Fig. S4. β4-integrin and GM130 localization is polarized in IKVAV-modified eBMs. (A) Peripheral:interior β4 integrin localization in IKVAV-modified eBMs. (B) Interior:Peripheral GM130 localization in IKVAV-modified eBMs. Statistical significance was tested by one-way ANOVA and post hoc multiple comparison tests for integrin intensity (p-value < 0.05). * indicates $p < 0.05$, ** $p < 0.01$, *** $p < 0.001$, **** $p < 0.0001$.

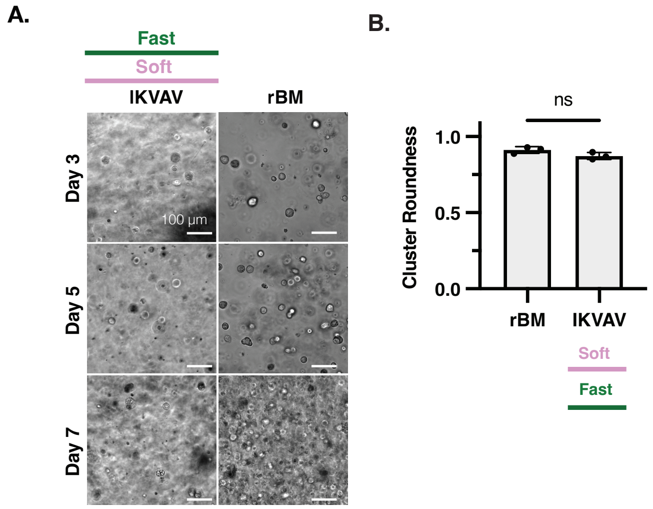

Fig. S5. PDM-350 patient-derived tumor organoids in IKVAV-modfied eBMs and rBMs. (A) Representative brightfield images of PDM-350 organoids in IKVAV-modified eBMs compared to rBM. (B) Quantification of cluster roundness from Panel A. All scale bars = 100 µm. Data are shown as mean ± SD of three biological replicates (n=3, the average of 20-50 images per replicate) unless otherwise indicated. An unpaired t-test was used for PDM-350 cluster roundness. If no statistical significance indicator bars are shown, there were no significant differences (p-value > 0.05). * indicates $p < 0.05$, ** $p < 0.01$, *** $p < 0.001$, **** $p < 0.0001$, ns= not significant.

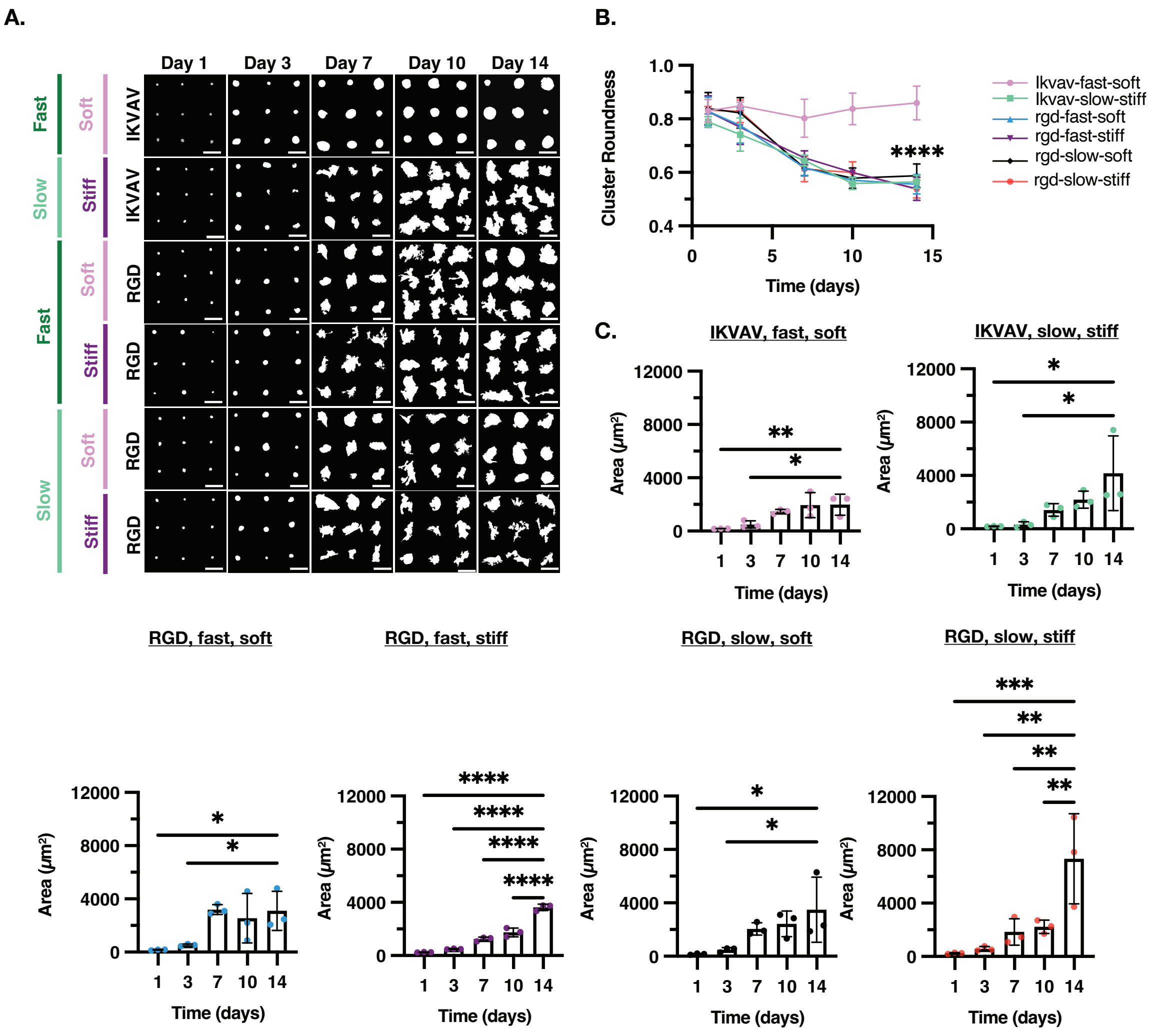

Fig. S6. Quantification of roundness and area over time in eBMs. (A) Representative outlines of MCF10As in modified-eBMs at Day 1, 3, 7, 10 and 14). Scale bars = 100 µm. (B) Cluster roundness throughout culture period for all eBMs.(C) Cluster area throughout culture period for all eBMs. Data are shown as mean ± SD of three biological replicates (n=3, 20-50 images per replicate) unless otherwise indicated. Statistical significance was tested by a Kruskal-Wallis test followed by Dunnett's multiple testing correction for cluster roundness, by one-way ANOVA and post hoc multiple comparison tests for cluster area (p-value < 0.05). * indicates $p < 0.05$, ** $p < 0.01$, *** $p < 0.001$, **** $p < 0.0001$.

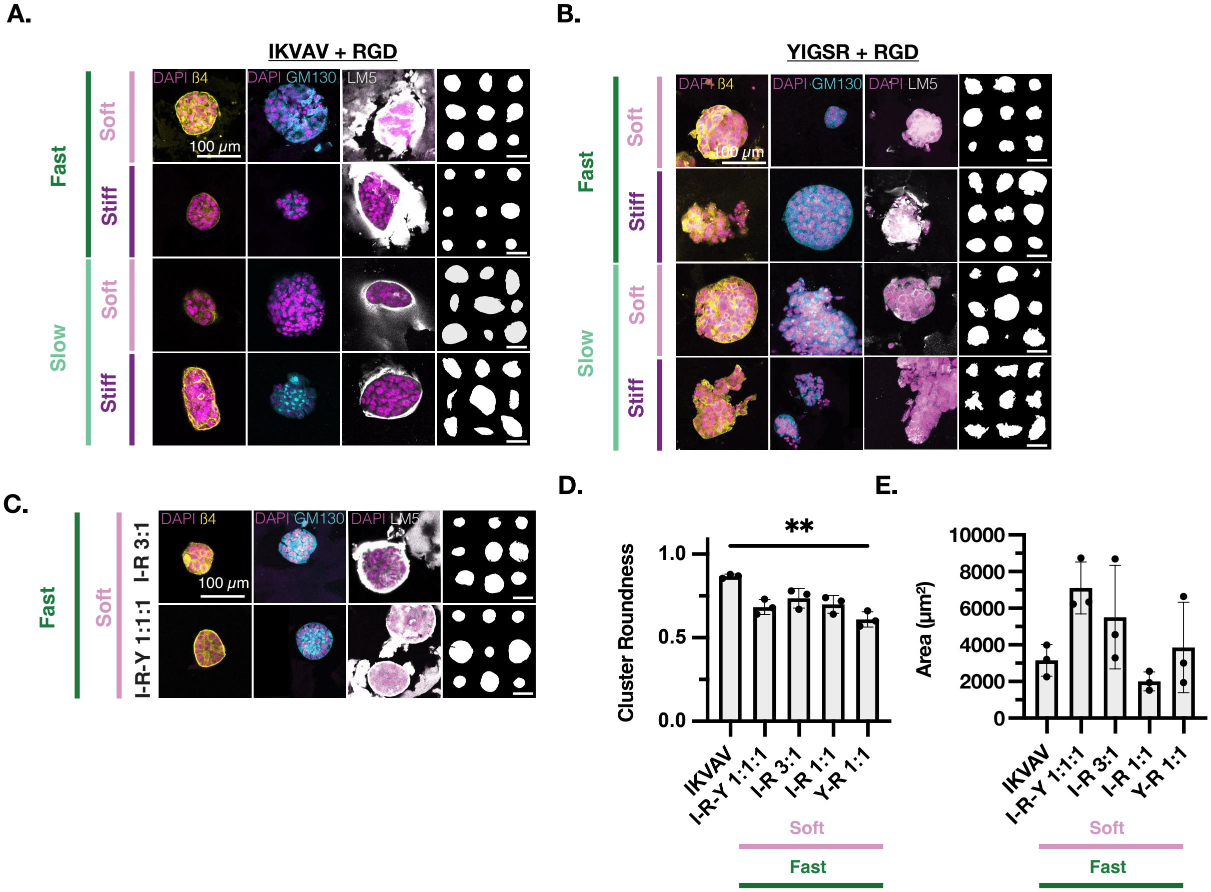

Fig. S7. RGD addition does not enhance acinar formation. (A) Representative outlines of MCF10As in IKVAV and RGD-modified eBMs. From left to right: DAPI/ β4-integrin, DAPI/GM130, DAPI/LM5 (332), and representative outlines. (B) Representative outlines of MCF10As in YIGSR and RGD-modified eBMs. From left to right: DAPI/ β4-integrin, DAPI/GM130, DAPI/LM5 (332), and representative outlines. (C) Representative outlines of MCF10As in mixed modified eBMs. From left to right: DAPI/ β4-integrin, DAPI/GM130, DAPI/LM5 (332), and representative outlines. (D) Roundness quantification and (E) area quantification of conditions compared to IKVAV-alone matrices. Scale bars = 100 µm. Data are shown as mean ± SD of three biological replicates (n=3, 20-50 images per replicate) unless otherwise indicated. Statistical significance was tested by a Kruskal-Wallis test followed by Dunnett's multiple testing correction for cluster roundness, by one-way ANOVA and post hoc multiple comparison tests for cluster area (p-value < 0.05). * indicates $p < 0.05$, ** $p < 0.01$, *** $p < 0.001$, **** $p < 0.0001$.

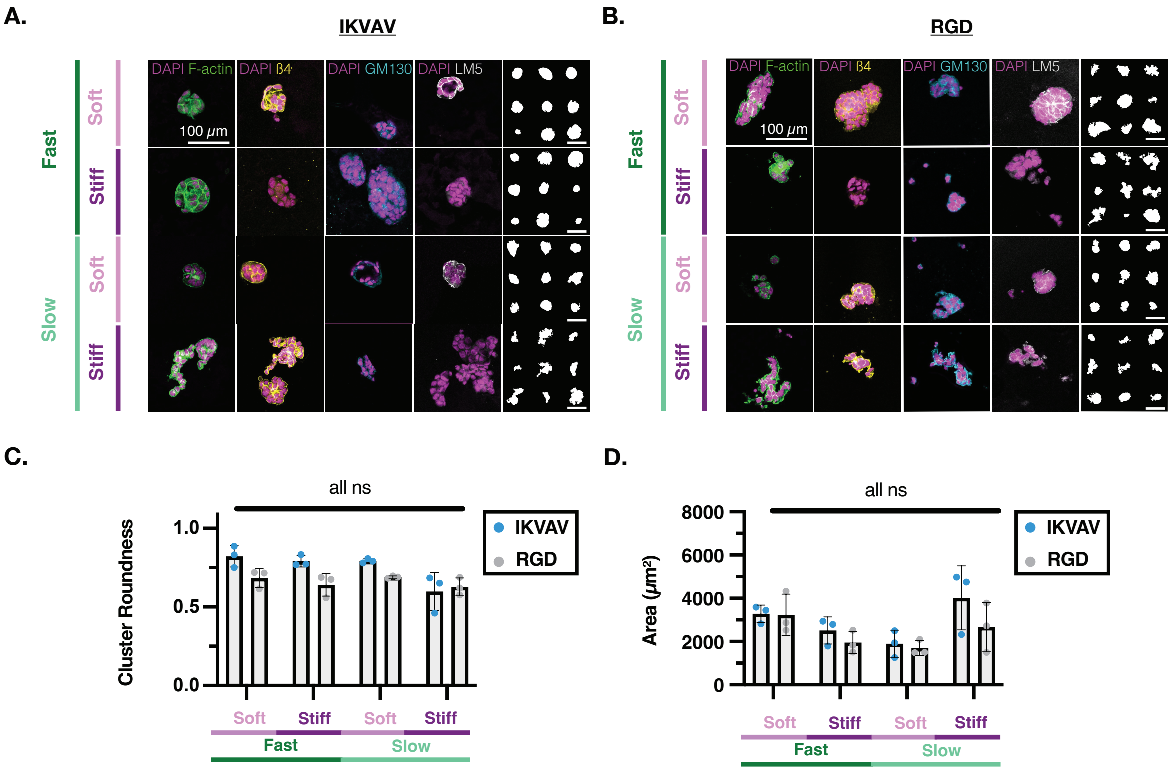

Fig. S8. MCF7s exhibit similar trends as MCF10As in modified eBMs. (A) Representative outlines of MCF7s in IKVAV-modified eBMs. From left to right: DAPI/F-actin, DAPI/β4-integrin, DAPI/GM130, and representative outlines of the respective condition. (B) Representative outlines of MCF7s in RGD-modified eBMs. From left to right: DAPI/F-actin, DAPI/β4-integrin, DAPI/GM130, and representative outlines of the respective condition. (C) quantified cluster roundness and (D) quantified cluster area. Scale bars = 100 μm. Data are shown as mean ± SD of three biological replicates (n=3, 20-50 images per replicate) unless otherwise indicated. Statistical significance was tested by a Kruskal-Wallis test followed by Dunnett's multiple testing correction for cluster roundness, by one-way ANOVA and post hoc multiple comparison tests for cluster area (p-value < 0.05). * indicates $p < 0.05$, ** $p < 0.01$, *** $p < 0.001$, **** $p < 0.0001$.

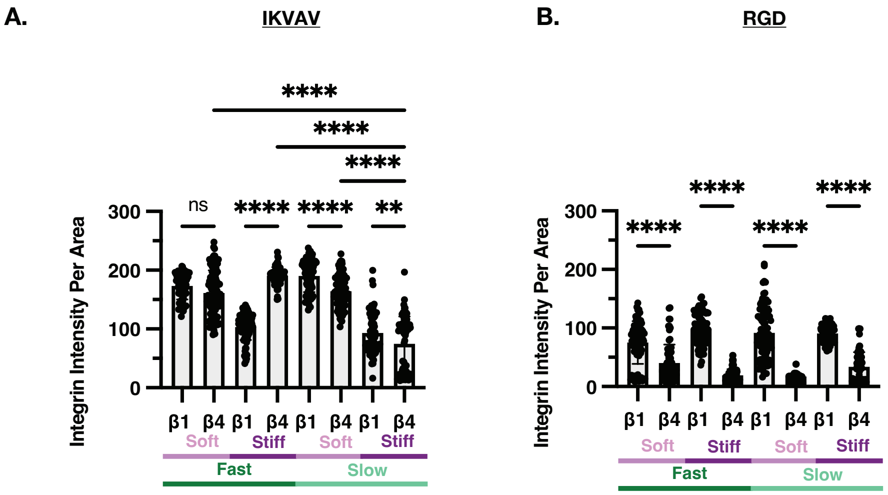

Fig. S9. IKVAV- and RGD-modified eBMs have differing integrin expression. (A) β1 and β4 integrin in IKVAV-modified eBMs. (B) β1 and β4 integrin in RGD-modified eBMs. Data are shown as mean ± SD of three biological replicates (n=3, 20-50 images per replicate) unless otherwise indicated. Statistical significance was tested by one-way ANOVA and post hoc multiple comparison tests for integrin intensity (p-value < 0.05). * indicates $p < 0.05$, ** $p < 0.01$, *** $p < 0.001$, **** $p < 0.0001$.

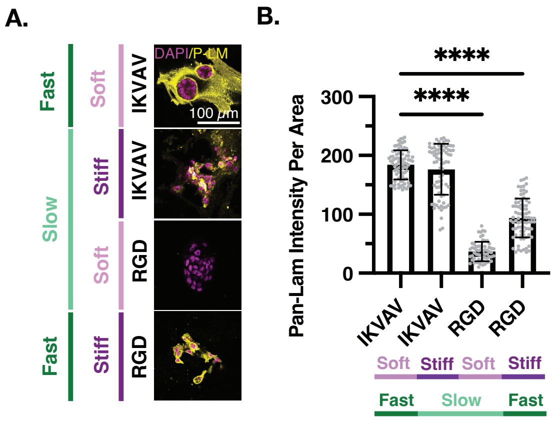

Fig. S10. Pan-laminin intensity is significantly higher in IKVAV-modified eBMs. (A) Representative confocal images of MCF10As encapsulated in IKVAV- and RGD-modified eBMs. DAPI/Pan-laminin. (B) Quantification of mean pan-laminin intensity per area from Panel A. All scale bars = 100 µm. Statistical significance was tested by one-way ANOVA and post hoc multiple comparison tests for pan-laminin intensity. * indicates $p < 0.05$, ** $p < 0.01$, *** $p < 0.001$, **** $p < 0.0001$.

	Peak ~5 ppm Integration	Peptide Peak Integration	Final peptide Concentration
Alginate (LMW) +low-IKVAV	31.1	1.2	156 μM
Alginate(LMW) +high-IKVAV	15.9	5.9	635 μM
Alginate(HMW) +high-IKVAV	36.4	36.1	834 μM
Alginate(HMW) +low-IKVAV	36.1	3.1	271 μM
Alginate (LMW) +high-YIGSR	61.8	2.1	252 μM
Alginate (LMW) +low-YIGSR	1276.0	2.2	25 μM
Alginate (HMW) +high-YIGSR	18.0	2.2	935 μM
Alginate (HMW) +low-YIGSR	171.8	3.4	295 μM
Alginate (LMW) +high-RGD	4.3	1.0	885 μM
Alginate (HMW) +high-RGD	1.7	1.0	2239 μM

Table S1. The integration of assigned 1H signals on IKVAV-modified alginate. The integration of assigned 1H signals on alginate (G-blocks, ~5.1 ppm) and peptide (IKVAV= 0.7–1.0, YIGSR=~7, RGD=~2 ppm) signals are shown, enabling the calculation of molar peptide concentration relative to the alginate monomer count. For example, Alg-CQAASIKVAV: 3 protons from Ile 3-Hγ, 3 protons from Ile 3-Hδ, and 12 protons from Val 2-Hγ, totaling 18 protons. For RGD, 8 protons (2x Pro Hβ/Hγ and 2x Arg Hβ/Hγ) were used. For YIGSR, 4 protons were used (2xTyr Hδ/Hε). The molar peptide concentration was calculated using the equation: $M_p=[71*M_a*I_1]/[M_h*I_5]$. $M_a = 2.67 \times 10^{-4}$ when using a 2% solution of alginate. M_h represents the number of protons analyzed for the given peptide (e.g. $M_h=18$ for IKVAV, $M_h=4$ for YIGSR and $M_h=8$ RGD). I_1 and I_5 correspond to the peptide peak(s) and the G-blocks peaks from alginate (~5.0 ppm), respectively.
